# Risk factors associated with under-five stunting, wasting, and underweight in four provinces of the Democratic Republic of Congo: analysis of the ASSP project baseline data

**DOI:** 10.1186/s12889-022-14842-x

**Published:** 2022-12-23

**Authors:** Joy Kinko Luzingu, Nancy Stroupe, Halimatou Alaofe, Elizabeth Jacobs, Kacey Ernst

**Affiliations:** 1 Department of Epidemiology and Biostatistics, Mel and Enid Zuckerman College of Public Health, The University of Arizona, 1295 N Martin Ave, Tucson, AZ 85724 USA; 2 IMA World Health, 1730 M Street, NW, Suite 1100, Washington, DC 20036 USA; 3 Department of Health Promotion Sciences, Mel and Enid Zuckerman College of Public Health, The University of Arizona, 1295 N Martin Ave, Tucson, AZ 85724 USA

**Keywords:** Prevalence, Undernutrition, Children, Socio-economic, Democratic Republic of Congo

## Abstract

**Background:**

Determining the magnitude and risk factors of undernutrition in a country that has one of the highest prevalence of undernutrition in the world is paramount for developing contextual interventions.

**Methods:**

This study used baseline data from the ASSP project to estimate prevalence of stunting, wasting, and underweight in four provinces of DRC. It involved 3911 children aged 0–59 months old and mother pairs. Height-for-age Z scores, Weight-for-height Z scores, and Weight-for-age Z scores were calculated and used to classify child stunting, wasting and underweight respectively, based on the 2006 World Health Organization (WHO) growth reference. Hierarchical logistic regressions were used to identify risk factors associated with stunting, wasting and underweight. All analyses were conducted using STATA 15.1, and statistical significance was set at *p* < 0.05.

**Results:**

The prevalence of stunting, underweight and wasting was 42.7%, 21.9% and 8.2% respectively. Increasing child’s age was a risk factor associated with stunting and underweight, while sex was not associated with the 3 indicators of undernutrition. Low levels of mother’s education, mothers working in the last 12 months prior to the survey, children living in the province of Kasai occidental, children born at a health facility, children perceived by their mothers to be born very small were associated with higher risks of stunting. Factors associated with underweight were children from the province of Kasai occidental, mothers who worked in the last 12 months prior to the survey, and children perceived to be born very small or small by their mothers. Children born to mothers aged 35–49 years and children breastfed in combination with drinking water were at higher risk of wasting.

**Conclusion:**

Prevalence of undernutrition in DRC is high. This study has identified certain modifiable risk factors associated with stunting, wasting and underweight. To reduce the burden of undernutrition in DRC, authorities should target factors at individual and community levels by improving women’s education, child feeding practices and promoting agriculture.

## Introduction

Undernutrition is a global problem that contributes to 45% of deaths among children under five years of age [1]. Sub-Saharan Africa (SSA) is the part of the world that harbors one-third of undernourished children, a deplorable situation that will possibly impede the development of this part of the continent if no action is taken [2]. A child who lacks appropriate nutrition may never attain their full mental and physical potential, hindering their academic and employment opportunities [3]. In terms of nations, undernutrition negatively influences their economic growth and prosperity due to the impairment of children’s long-term physical, mental and emotional development [4]. The Democratic Republic of Congo (DRC), has a very young population [5], and one of the highest prevalence of undernutrition in the world [6].

Undernutrition is measured using three primary indicators: First, stunting is an indicator of chronic malnutrition, caused by sustained insufficient nutrient intake and repeated infections. Second, wasting is an indicator of acute malnutrition, resulting from acute food shortage and disease. Finally, underweight combines information of chronic and acute malnutrition. While the effects of stunting include retarded motor development and impaired intellectual development which are irreversible, wasting is a stronger predictor of mortality and demands urgent reaction [7]. According to the last 2013–14 Demographic and Health Survey (DHS) of DRC, there were 43% of children under five years suffering from chronic malnutrition (23% with severe chronic malnutrition), 8% of children suffering from acute malnutrition (3% having the severe form), and 23% suffering from underweight (7% in the severe form) [8]. In DRC, the prevalence of child stunting remains high. At 41.8% in 2017–18, it has not declined significantly since 2001 when 44% of Congolese children were stunted. On the other hand, wasting in DRC has substantially declined since 2001, reducing from 15.9% to 6.1% in 2017–18 [9]. However, these figures are still alarming since the World Health Organization (WHO) suggested that stunting prevalence over 30% is considered severe, and wasting over 5% is an indicator of food insecurity [10]. These findings suggest an urgent need for interventions to reduce the burden of undernutrition in the country.

According to the United Nations International Children’s Emergency Fund’s (UNICEF) conceptual framework, determinants of undernutrition are categorized into immediate (disease and dietary intake), underlying (household and environmental factors such as food security, feeding practices, access to safe drinking water and toilet, and healthcare) and basic causes (geographical region, wealth, sociocultural, economic and political context) [6]. Many studies conducted in SSA have found sex of the child, age, birth weight, birth interval, number of children under-five in the household, maternal education, breastfeeding status, household wealth index, unimproved water, poor hygiene and sanitation as factors associated with risk of undernutrition among children under-five years of age [11–14]. Risk factors for undernutrition in DRC have been described in some studies. For example, Kismul et al. found that male children, age older than 6 months and preceding birth interval less than 24 months were risk factors associated with stunting [15]. Using the 2001 DRC Multiple Indicators Cluster Survey (MICS), a study found higher risks of undernutrition among children born from less educated mothers [16]. Using 2007 DRC DHS data, another study identified that stunting was more prevalent in rural areas compared to urban ones [17]. More recently, McKenna et al., found no association between women’s decision-making power and children’s stunting and wasting [18].

Despite the high prevalence of undernutrition among children in DRC, there is limited evidence to guide decision-making and resource allocation for interventions, especially at the local level. Population-based cross-sectional surveys, such as the DHS and MICS, have been used worldwide to evaluate the prevalence of different forms of malnutrition in a country or through cross-country comparisons [15, 19–22]. However, country-level estimates may be less helpful in informing the local, community-based interventions common in resource-limited settings. Thus, administering available surveys at the local level offers a solution for providing rapid, local estimates of health burdens in at-risk populations. In addition, to our knowledge, studies that investigated the risk factors of undernutrition among children in DRC lack a theoretical framework or rationale that informs the selection of variables that construct the concept of risk factors of undernutrition. Finally, few recent studies have examined a breadth of risk factors associated with the three indicators of undernutrition in the DRC. Therefore, to address the knowledge gap, this study aims to estimate the prevalence of stunting, wasting, and underweight in four provinces of DRC and assess their relationship with child, maternal, and household level variables. Identifying risk factors of undernutrition in this population using the conceptual framework that aligns with data available in the “Accès aux soins de santé primaire (ASSP)” project may help decision-makers formulate evidence-based policies targeting the reduction of undernutrition in DRC.

## Methods

### Study site

**Fig. 1:**
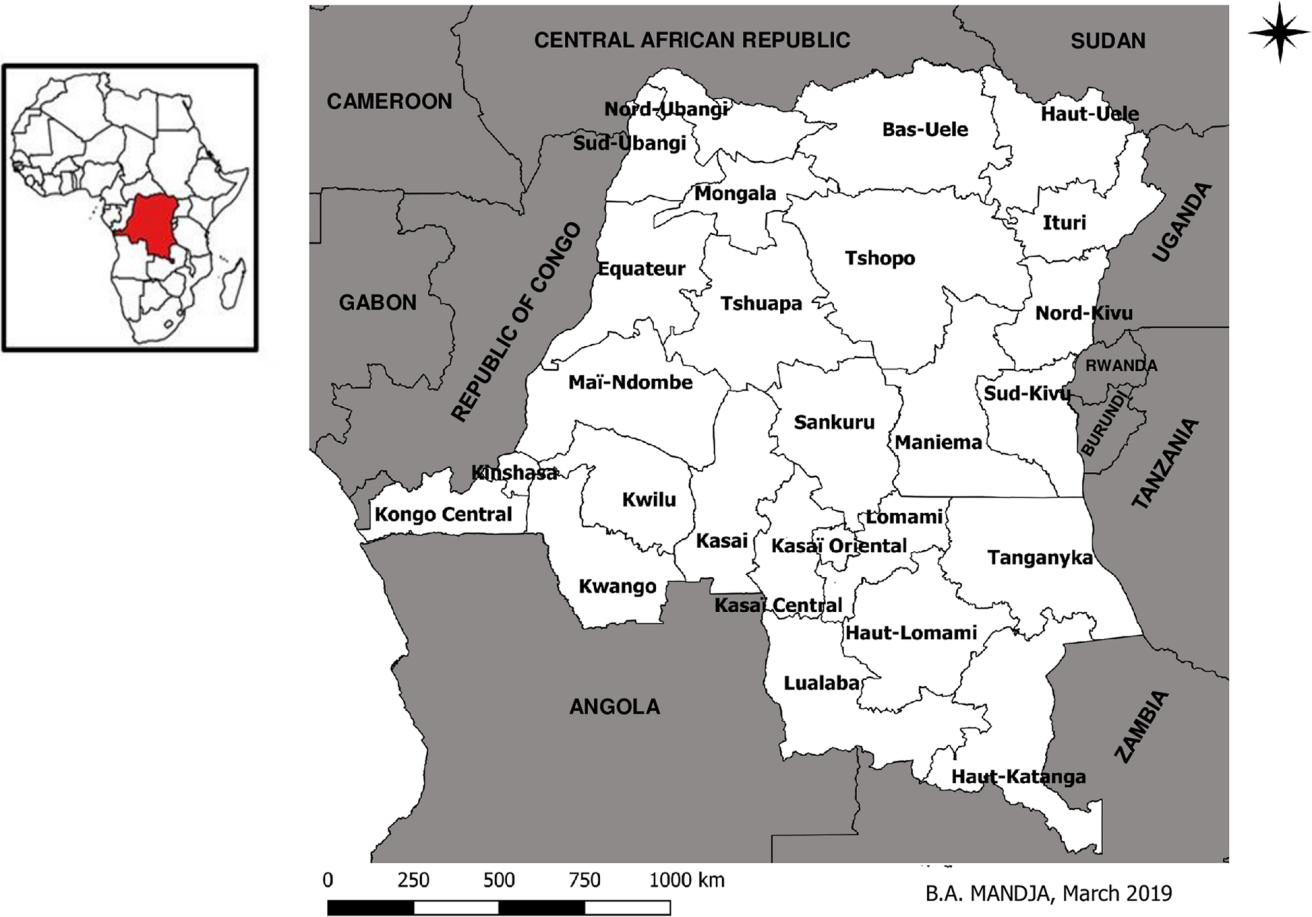
Administrative map of the DRC including 26 new provinces and bordering countries (Source: Mandja, et al. The score of integrated disease surveillance and response adequacy (SIA): a pragmatic score for comparing weekly reported diseases based on a systematic review. BMC Public Health. 2019; 19(1): 1–14[24])

The DRC is the second-largest country in Africa, with a total population of 89,561 million inhabitants [23]. It is administratively subdivided into 26 provinces (Fig. 1) [24].

**Fig. 2:**
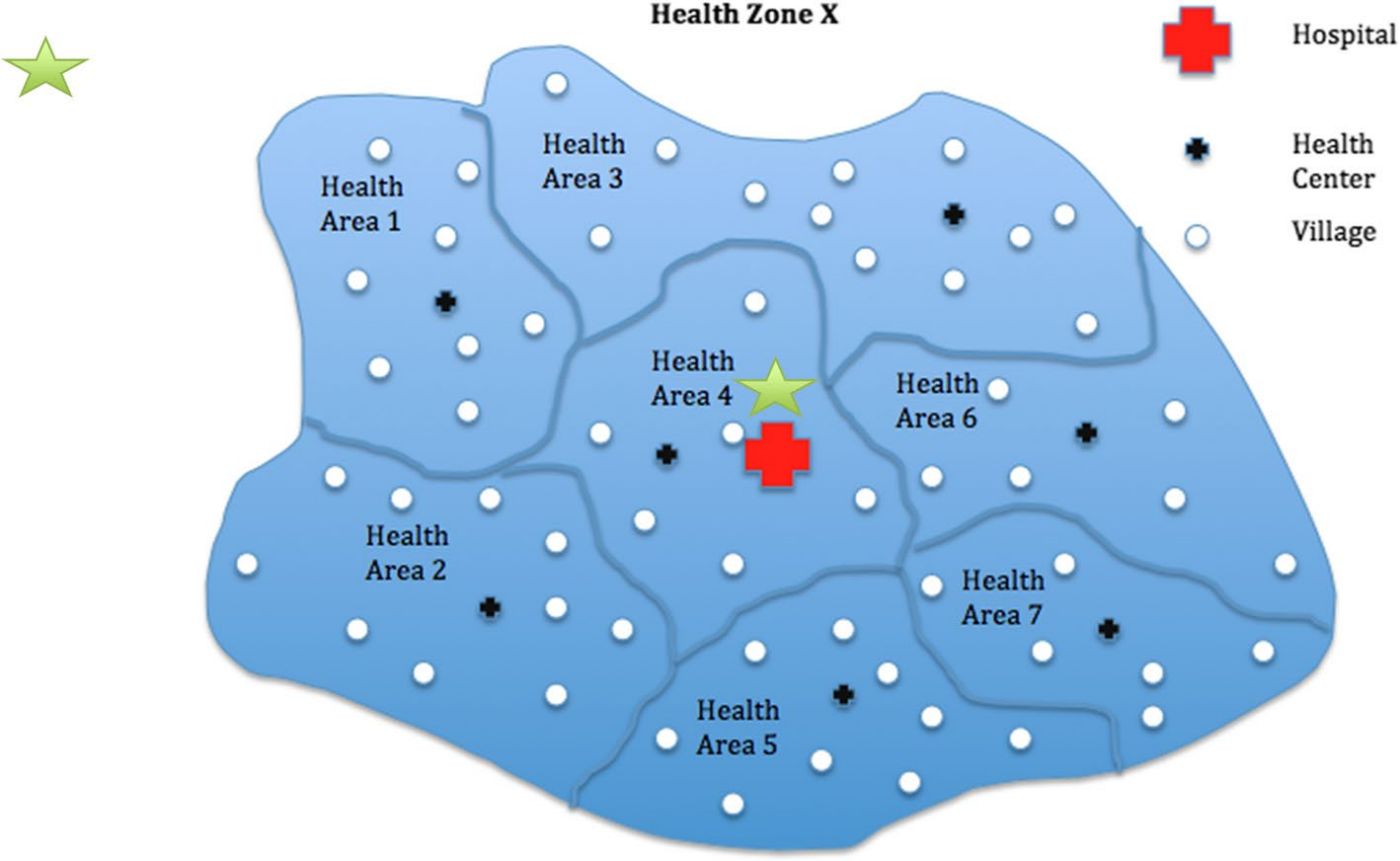
Organization of health zones in the Democratic Republic of Congo (Source: Research Protocol Evaluation of the Impact of the ASSP Project in the Democratic Republic of Congo)

The DRC health system is organized in three levels. At the implementation level, it is divided into 516 health zones (HZs). Each HZ is further divided in health areas (HAs) (Fig. 2). HZ and HA, including the general hospital and health centers are led by the health zone management team (*Equipe Cadre de la Zone de Santé* (ECZS)) located at the Central Office of the Health Zone (*Bureau Centrale de la Zone de Santé* (BCZ)). Theoretically, each HZ harbors one general reference hospital and is supposed to serve anywhere from 100,000 people in rural areas to 200,000 people in urban areas. Additionally, each HA has at least one health center and serves on average 17 villages in rural areas or neighborhoods in urban areas. At the intermediate level, in charge of logistic and technical support, it comprises provincial health departments in number of 26. The central level plays a normative role [25].

**Fig. 3:**
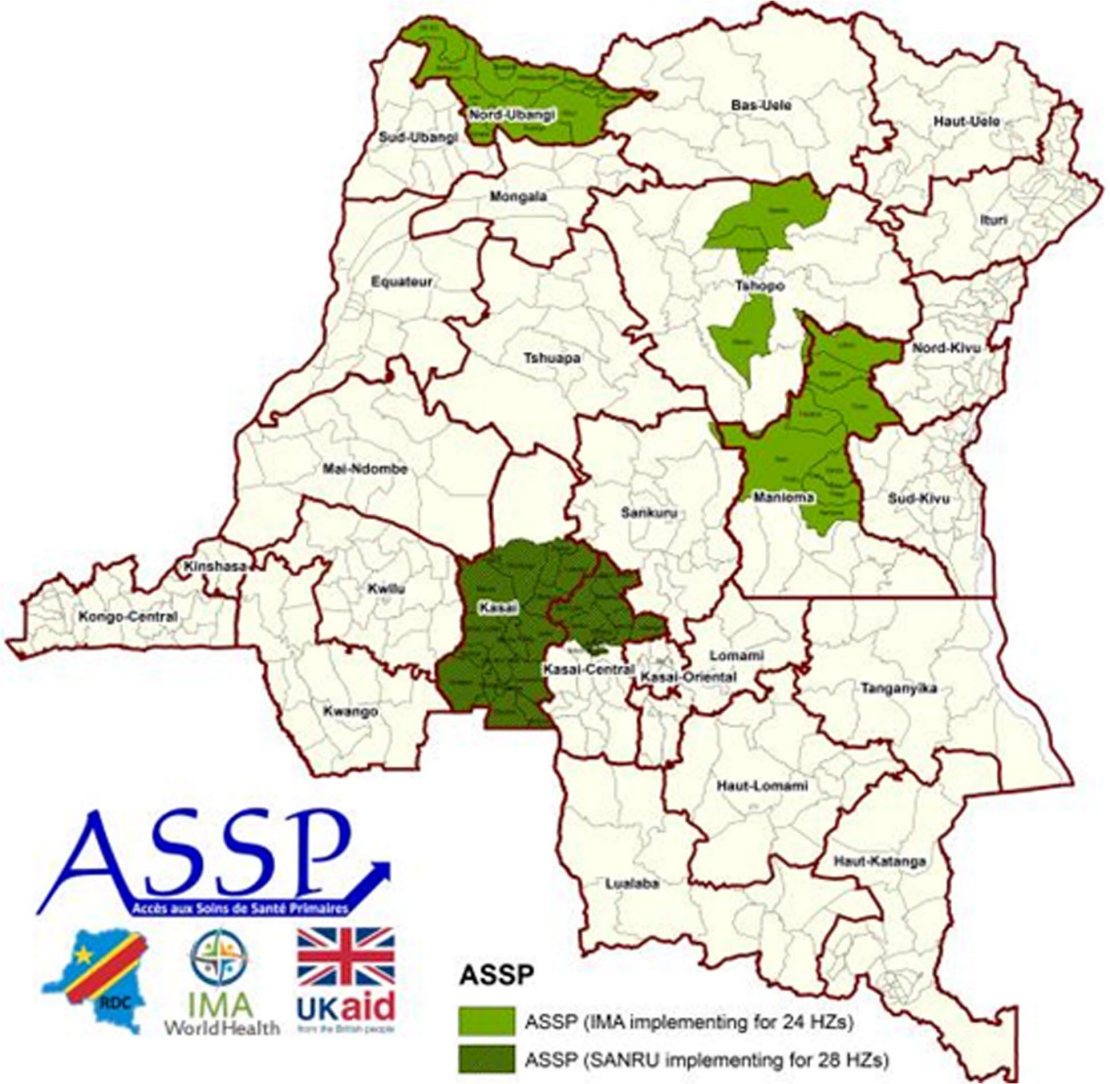
ASSP Project Areas in the Democratic Republic of Congo (image courtesy of IMA World Health)

This study uses data from the baseline evaluation survey a project funded by the United Kingdom (UK) Department for International Development (DFID) and implemented by IMA World Health in 52 HZs of 5 provinces of the DRC, namely North Ubangi (formally Equateur), Tshopo (formally Oriental province), Kasai and Kasai central (formally Kasai occidental) and Maniema. The project was titled “Accès aux soins de santé primaire (ASSP)” and aimed at reducing morbidity and mortality in women and children under five while strengthening the country’s health system. In March of 2015, a presidential ordinance was issued calling for a new administrative configuration of DRC’s provinces, such that the existing 11 provinces were split into 26. The former Kasai Occidental was split into 2 provinces, Kasai and Kasai Central, both of which contained ASSP health zones; Equateur was divided into 5 provinces, with ASSP health zones located in North Ubangi; Province Orientale was split into 4 provinces with ASSP health zones located in Tshopo; Maniema remained one province. The number of HZs per province supported by the ASSP project differed greatly (17 in Kasai, 11 in Kasai Central, 10 in Maniema, 4 in Tshopo and 11 in North Ubangi) (Fig. 3). Those targeted health zones were chosen for two reasons: 1) they were considered to have weak health systems, and/ or 2) DFID had started to provide support to the health zones under a preceding project and wanted to continue that assistance. For consistency with the way data were collected in 2014 in the four former provinces, before the presidential ordinance, we considered those four provinces and named them accordingly in this study: Equateur, Kasai occidental, Maniema and Orientale.

### Study design and source of data

**Fig. 4:**
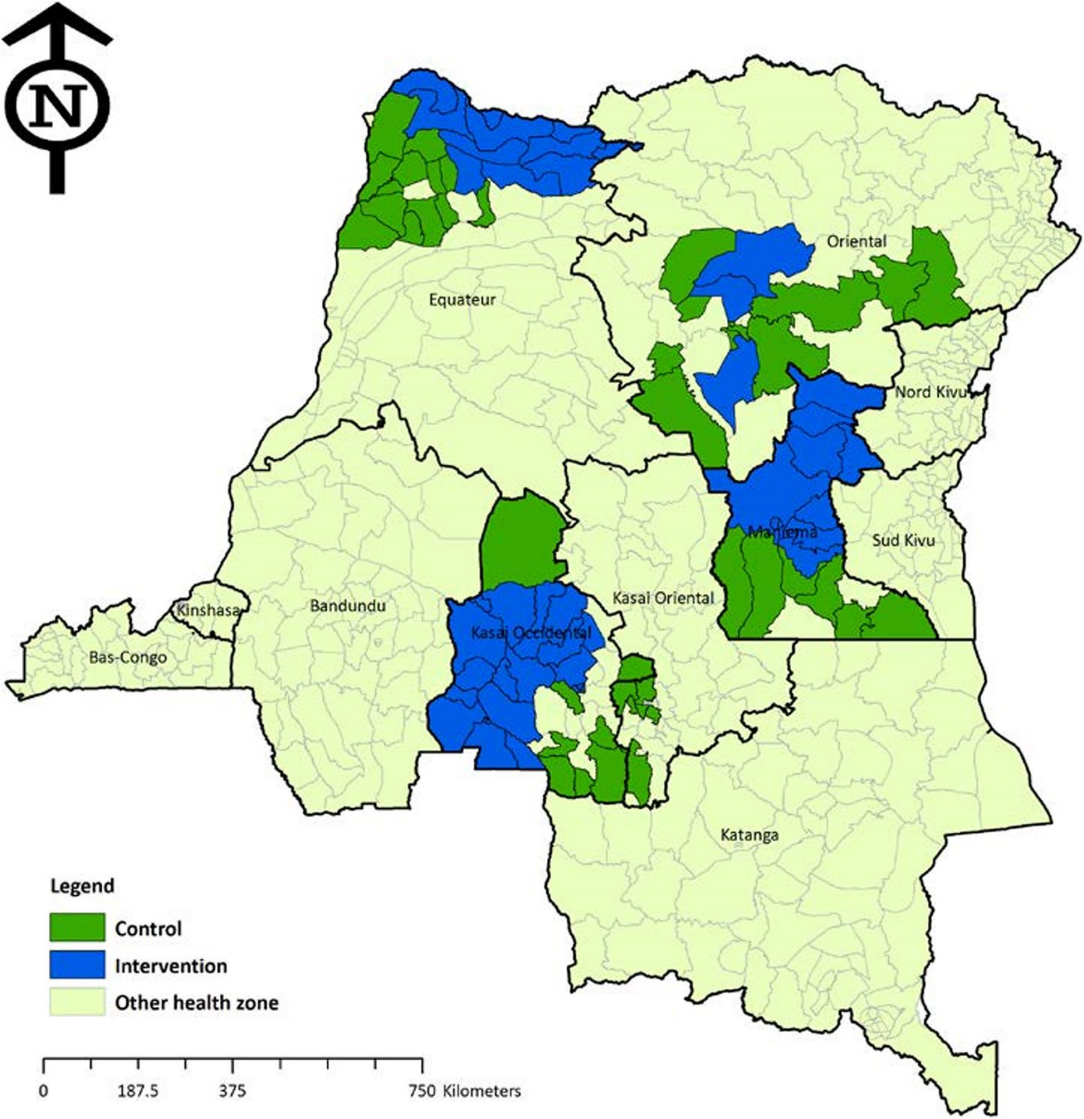
Map of ASSP and matched comparison health zones samples in baseline evaluation. (Source: Report Baseline survey women and households, ASSP)

We conducted a secondary analysis using baseline data from a cross-sectional population-based study that was conducted in 2014 by the Tulane University School of Public Health and Tropical Medicine. The baseline survey included 4 provinces, namely the Kasai occidental, Maniema, Equateur, and Oriental provinces. The provinces were divided into three sampling areas; the first sampling zone consisted of HZs of provinces of Maniema and Orientale, the second comprised HZs of the Kasai occidental province and the third included in the Equateur province. Matched comparison groups consisting of randomly selected villages within matched HAs outside ASSP supported HZs that did not receive ASSP intervention packages were also selected (Fig. 4).

Per the research protocol, at the intervention sampling areas, a two-stage sampling method was used to collect data at baseline. At the first stage, a full roster of villages was separated into three sampling areas (3 strata). Then, 35 villages (primary sampling units or clusters) were selected for each sampling area, resulting in 105 villages selected (35 villages per sampling area). At the second level, 20 households were selected from each village to attain the desired total sample of 700 households in each sampling area. For the comparison groups, a three-stage sampling design was used because a complete roster of villages with population estimates was not available. At first the stage, a comparison group HA was matched to an HA containing a village in the corresponding intervention area using a list of HAs with population estimates provided by the health authorities. Matching criteria were geographical location (same province or geographic area but close enough to the ASSP assisted HZs), HA population size (greater than or less than 5,000 persons), HA urban/rural status and HZ vaccination coverage (lowest, low, second high, highest). Then, 35 of those matched HAs were selected within each comparison group, meaning that 105 comparison HAs were selected. At the second level, a village was randomly selected from the detailed list of HAs in the comparison area. At the third level, after enumerating households the same way it was done for the intervention areas, 20 households were systematically selected to reach the desired sample size of 700 households in each comparison group. Within each household selected, the household’s head and women of reproductive age (15–49 years) were interviewed. All persons who slept in the household a day before the survey and all household members were considered household residents. Information on all children under five years was obtained from mothers when possible. When the mother was not present, information on children was obtained from the child’s caregiver, including vulnerable children, orphans, and child-headed households.

**Fig. 5:**
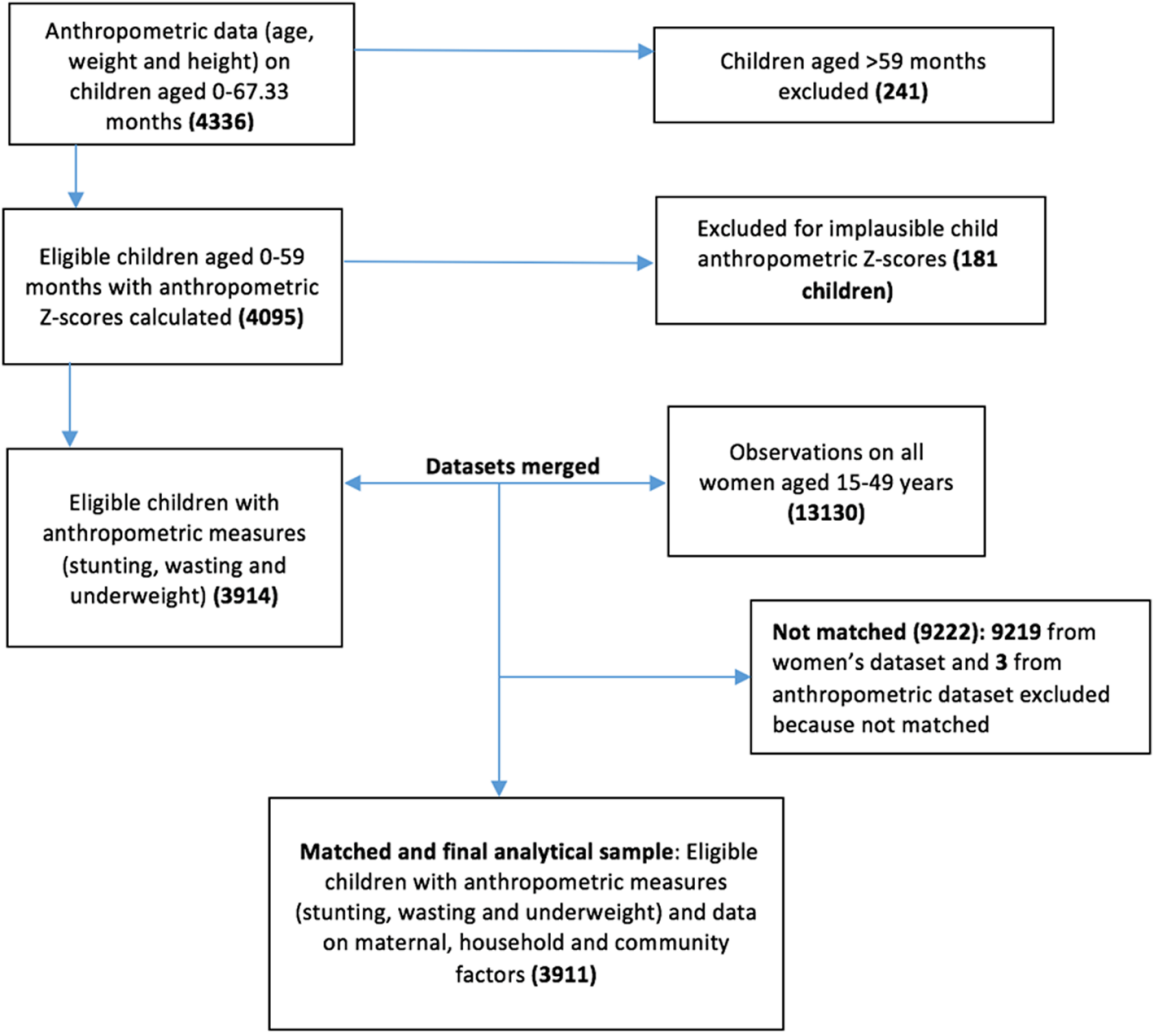
Participant flow diagram

Of the 2069 households selected in the intervention areas and 2109 households in the matched comparison areas, 98.6% of interviews were successfully completed. This yielded anthropometric data for 4336 children aged 0–67.33 months (anthropometric dataset). From those, we excluded 241 children aged > 59 months, resulting in 4095 children aged 0–59 months remaining in the anthropometric dataset. Then, we calculated anthropometric Z scores for all those children and excluded 181 of them who had biologically implausible anthropometric Z-scores, that is Weight-for-height Z-scores (WHZ) < -5 or > 5 (63 children), Height-for-age Z-scores (HAZ) < -6 or > 6 (103 children), Weight-for-age Z-scores (WAZ) < -6 or > 5 (3 children) and Body mass index Z-score (Z-BMI) < -5 or > 5 (12 children) based on the WHO flagging convention [26]. We then merged the anthropometric dataset which contained 3914 remaining children with the women’s dataset that contained 13,130 observations from all women/caregivers aged 15–49 years, using the unique children identification variable (‘cmergeid’). In total, 3911 observations matched and constituted our final analytical sample (Fig. 5).

### Measures

#### Primary outcome

The primary outcome of this study was undernutrition defined in terms of stunting, wasting, and underweight, and categorized as binary variables. To define the primary dependent variable, data on age were obtained during interviews with mothers for children aged under 60 months by asking the month and year of birth of the child. Data on weight and height were obtained using standard scales and height measurement tools. Using the WHO Child Growth Standard STATA igrowup package, we obtained height-for-age, weight-for-height, and weight-for-age Z scores for the study participants (HAZ, WHZ and WAZ, respectively). We classified nutritional status according to the 2006 child growth standard of the WHO [27]. Any child below -2SD of reference height for their specific age was ‘stunted’ (HAZ < -2 SD), and any child below -2SD of reference weight for their specific height was ‘wasted’ (WHZ < -2 SD), while any child below two standard deviations (-2SD) of reference weight for their specific age was ‘underweight’ (WAZ < -2 SD).

**Fig. 6:**
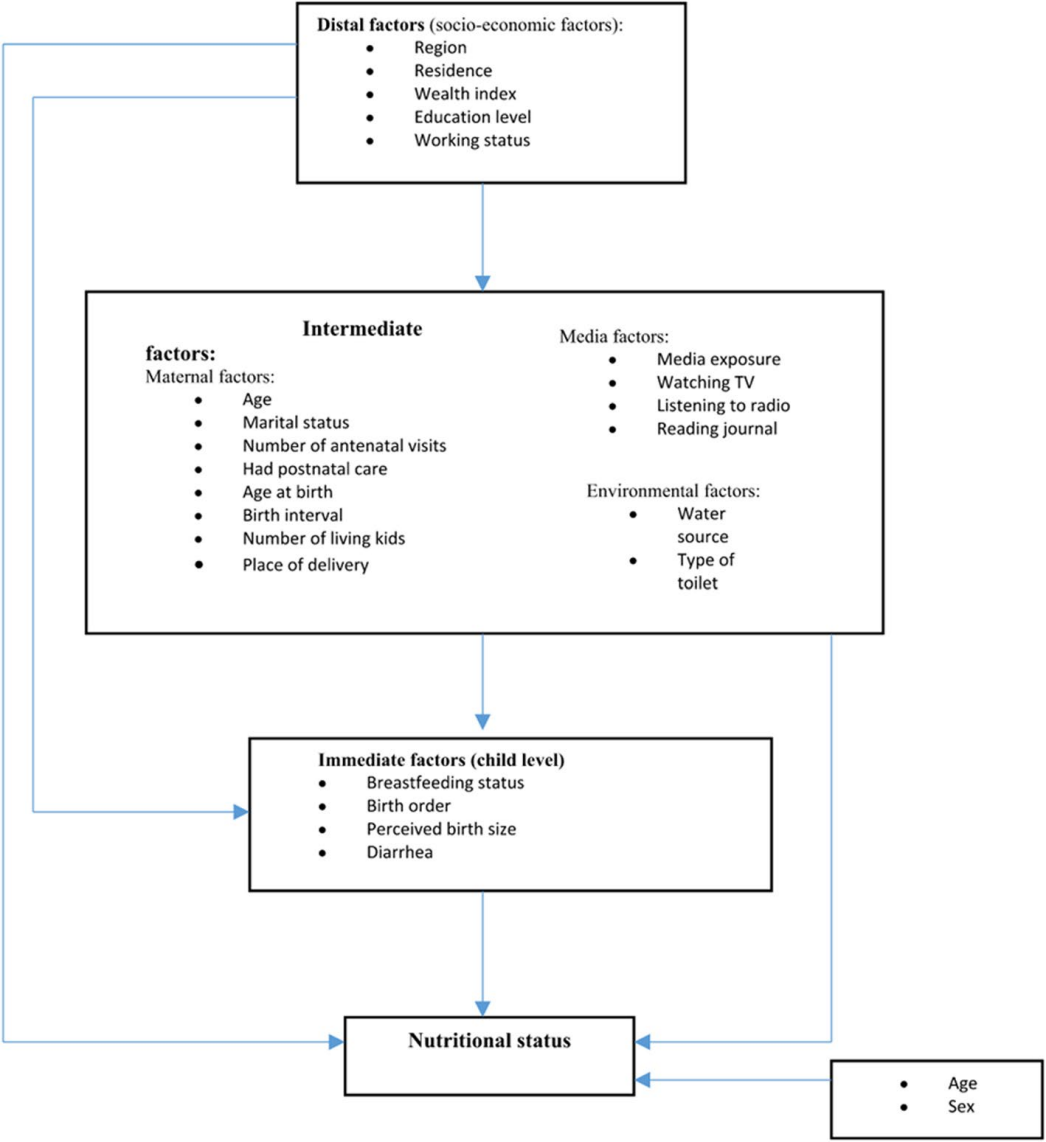
Conceptual framework of the determinants of undernutrition in children (source: modified from Hien & Hao, 2009 [28] and UNICEF framework [6])

#### Independent variables

We selected covariates to include in this study based on the UNICEF framework [6] and previous studies that have identified risk factors of undernutrition in children [11, 15, 19, 22]. Then, we modified the conceptual framework from Hien and Hao [28] based on the UNICEF framework and hypothesized that there are hierarchical relationships between three levels of risk factors of undernutrition. Indeed, it was demonstrated that in developing countries, child health is determined by a large set of factors. Most diseases in such societies may be related to poverty caused by lack of or unfair distribution of resources. In epidemiology, poverty is often assessed using family income, parental education level or possession of some household assets. However, these factors do not cause disease directly and are qualified as distal factors. They more often act through a series of interrelated proximate factors qualified as intermediate factors. Therefore, it may happen that when distal factors are inappropriately adjusted for proximate factors, there is a reduction or elimination of their effects in the model [29]. Our conceptual framework is based on the premise that distal factors may determine children’s undernutrition either directly or indirectly through some inter-related intermediate and immediate factors except child’s age and sex. In turn, those intermediate factors exert their effect on undernutrition either directly or through some immediate factors. Immediate factors, in turn, may affect undernutrition directly. In this model, the three conditions for a factor to be a confounder are respected when a factor is associated with the exposure, is a predictor of the outcome, and is not a mediating factor, that is, it is not in the causal pathway going from the risk factor to the outcome. Thus, we assessed the effect of distal factors in model 1. Then, we added the intermediate factors to the model, and we assessed their effect in presence of distal factors which respected the conditions for confounding factors, leaving the possibility to obtain the effect of intermediate factors controlled for gender and sex (model 2). Finally, we entered immediate factors in the model, and we assessed their effect controlled for gender and sex in presence of both confounding factors, distal and intermediate variables.

The distal factors we included in our study were: the province of residence (Kasai occidental, Equateur, and Maniema/Province Orientale), place of residence (rural or urban), mother’s highest education level (none, some primary, complete primary and secondary and higher), mother’s working status (not worked in 12 past months, not currently working but worked in last 12 months, and currently working), and household wealth index. The wealth index was computed according to the Demographic and Health Survey (DHS) guidelines to generate a composite measure of a household’s cumulative living standard [30]. The composite measure was built by aggregating data on household’s productive and non-productive assets and other characteristics. Variables related to household’s ownership of selected goods (e.g., vehicles, livestock, agricultural land, television, refrigerator), materials used for housing construction and main house features (e.g., persons per sleeping room, arrangement of rooms, cooking fuel, electricity) and types of drinking waterand sanitation facilitations were selected... The generated score was weighted using the principal components analysis method and categorized into five wealth quintiles ranging from least wealthy (1 = low) to wealthiest (5 = high).

The intermediate factors we included in this study were mother’s age in years (15–24, 25–34, 35–44, 45–49), mother’s marital status (married/in a union, never married, divorced/ separated/ widowed), number of antenatal visit during the most recent pregnancy (none, 1–3, 4 + , don’t know), mother’s age at birth in years (less than 20, 20–34, 35–49), the birth interval in months (first birth, 0–23, 24–47, 48 +), number of living children in the household (1–2, 3–4, 5 +), place of delivery (home, health facility), postnatal care within 42 days of delivery (no, yes), exposure of the mother to media (no media exposure, some media exposure), mother listening to radio at least once a week (no, yes), mother watching television (TV) at least once a week (no, yes), mother reading a journal at least once a week (no, yes), type of drinking water source and type of toilet. Types of drinking water source were either improved or unimproved. Drinking water was defined as water used for domestic purposes, drinking, cooking, and personal hygiene. Improved drinking water was defined as a source that, by nature of its construction, adequately protects the water from outside contamination, in particular from fecal matter (examples: piped household water connection, public standpipe, borehole, protected dug well, protected spring, rainwater collection). Unimproved drinking water sources comprised unprotected dug well, unprotected spring, surface water (river, dam, lake, pond, stream, canal, irrigation channel), vendor-provided water (cart with small tank/drum, tanker truck), bottled water, and tanker truck water. Type of toilets were either improved/not shared or unimproved/shared. Improved toilets included facilities that hygienically separate human excreta from human contact such as those with sewer connections, septic system connections, pour-flush latrines, ventilated improved pit latrines and pit latrines with a slab or covered pit. Shared toilets were otherwise-acceptable improved sanitation facilities that are shared between two or more households. They included public toilets and were not considered improved. Unimproved toilets were defined as those that did not ensure a hygienic separation of human excreta from human contact and included pit latrines without slabs or platforms or open pit, hanging latrines, bucket latrines, open defecation in fields, forests, bushes, bodies of water or other open spaces, or disposal of human feces with other forms of solid waste.

The immediate factors included birth order (first born, 2–3, 4–5, 6 +), diarrhea episodes in the last 2 weeks before the survey (with diarrhea being defined as 3 or more loose or watery stools in a 24-h period), breastfeeding status of the youngest among children aged 0–23 months (not breastfeeding, exclusively breastfeeding, breastfeeding + water, breastfeeding + supplement) and perceived birth size as a proxy of birth weight. In fact, it was shown that perceived birth size is a good proxy for birth weight mostly in developing countries where many children are not weighed at birth [31] Fig. 6. 

### Statistical analysis

We performed statistical analyses with STATA version 15.1 using the “svyset” and “svy” commands to account for the design structure and weights in the ASSP dataset, and we considered *P* < 0.05 to be statistically significant. All statistical tests were two-sided.

For our descriptive statistics, we first presented participants by estimating unweighted frequencies and weighted percentages of categorical covariates, and we determined the prevalence of undernutrition according to participants characteristics using the Chi-square test Then, we presented the prevalence of the three undernutrition outcomes.

To assess the association between each undernutrition outcome and characteristics of children, we used three different logistic regression models for each of the outcome variables for children aged 0–23 months (using the subpopulation option) and for overall children aged 0–59 months. We used logistic regression to describe the association between binary outcome variables and a set of predictor variables. First, we assessed the independent association of the distal factors with each form of undernutrition in the absence of intermediate and immediate factors (model 1). Second, we fitted distal factors with intermediate factors to assess the association between intermediate factors and undernutrition adjusting for the confounding effect of distal factors (model 2). Finally, we fitted distal, intermediate, and immediate factors to assess the relationship between immediate factors and undernutrition adjusting for the confounding effects of distal and intermediate factors and the independent association between immediate factors and undernutrition (model 3). We included the variable breastfeeding status of the youngest among children aged 0–23 months as an immediate factor in model 3 only for children aged 0–23 months. We considered age and sex of the child as control variables and maintained them in all models. We considered child’s age and sex (male or female) as control variables for this study because they might work independently or through the distal, intermediate or immediate factors [15]. Age of children in months was categorized as 0–5, 6–11, 12–23, 24–35, 36–47 and 48–59 months.

We implemented this model fitting process by following Victory, Huttly et al.’s recommendations [29].

Although “stunting”, “wasting” and “underweight” are three different forms of undernutrition, we used the same covariates for all models so that we could make a comparison if there were common significant factors that can influence children’s nutritional status, and therefore propose recommendations to alleviate that consequence.

We tested collinearity at the end of each model with variance inflation factor (VIF) and all VIF we found were well below the maximum value of 10, indicating the absence of association between independent variables. We also tested the goodness-of-fit of each model using the “svylogitgof” command developed for a logistic regression model fitted using survey sample [32], and reported the F-adjusted mean residual test and *p*-value in the results tables. We reported adjusted odds (aOR) ratios and associated 95% confidence intervals (95% CI) for all risk factors.

## Results

### Sociodemographic characteristics of study participants

Sociodemographic characteristics are presented in Table 1. Of the 3911 children aged 0–59 months included in this study, 14.6% were under 6 months of age. The distribution of children by sex was similar (50.1% of girls and 49.9% of boys). The majority of children were perceived by their mothers to be born at an average size or more (90.1%), at a health facility (64.5%), born to married women (94.9%), lived in rural areas (89.9%), and in houses without improved water sources (76.6%) or improved toilets (96.2%). Only 3.4% of children had mothers with secondary and higher levels of education and 80.5% of children had mothers who were working at the time of the interview. Nearly 30% of children had mothers who did not have antenatal visits during their latest pregnancy, 81.1% of children had mothers who had no media exposure, and 15.7% of children had diarrhea in the 2 weeks prior to the survey.

**Table 1:** Prevalence of undernutrition according to demographic and socio-economic characteristics of children under five in DRC, ASSP 2014

Characteristics	Total children n (%)	Prevalence % [95% CI]		
		**Stunting**	**Wasting**	**Underweight**
**Child’s age in months**		*P* < 0.001	*P* = 0.0069	*P* < 0.001
- 0–5	516 (14.6)	19.5 [13.2; 28.0]	9.4 [4.9; 17.3]	9.0 [4.5; 17.06]
- 6–11	416 (8.9)	22.7 [16.0; 31.3]	17.0 [11.4; 24.5]	18 [12.2; 25.7]
- 12–23	809 (22.0)	40.5 [31.3; 50.4]	9.4 [5.4; 15.7]	19.9 [14.8; 26.2]
- 24–35	797 (18.5)	49.5 [42.2; 56.9]	8.3 [5.6; 12.2]	29.4 [23.8; 35.6]
- 36–47	729 (20.3)	55.9 [47.2; 64.3]	4.7 [2.6; 8.4]	25.8 [19.6; 33.1]
- 48–59	644 (15.7)	53.7 [45.1; 62.0]	4.5 [2.5; 8.2]	25.3 [20.1; 31.5]
- Total	3911 (100)	42.7 [37.3; 48.3]	8.2 [6.2; 10.6]	21.9 [18.5; 25.8]
**Child’s sex**				
		*P* = 0.2120	*P* = 0.1578	*P* = 0.3100
- Male	1966 (49.9)	44.1 [39.4; 48.8]	9.30 [6.6; 13.0]	22.96 [18.8; 27.7]
- Female	1945 (50.1)	41.3 [34.7; 48.3]	7.02 [5.1; 9.6]	20.87 [17.3; 25.0]
- Total	3911 (100)	42.7 [37.3; 48.3]	8.16 [6.2; 10.6]	21.92 [18.5; 25.8]
***Distal factors***				
**Province**				
		*P* = 0.0189	*P* = 0.5623	*P* = 0.0090
- Equateur	1304 (17.4)	40.5 [37.0; 44.0]	7.19 [5.6; 9.2]	20.63 [17.7; 23.6]
- Kasai occidental	1362 (54.6)	48.5 [42.3; 54.8]	7.69 [5.2; 11.2]	26.30 [22.0; 31.1]
- Maniema/Orientale	1245 (28)	32.7 [22.8; 44.4]	9.68 [5.4; 16.7]	14.16 [8.8; 22.1]
- Total	3911 (100)	42.7 [37.3; 48.3]	8.16 [6.2; 10.6]	21.92 [18.5; 25.8]
**Residence**				
		*P* = 0.3885	*P* = 0.5766	*P* = 0.1035
- Urban	455 (10.1)	38.2 [27.8; 49.8]	6.4 [2.5; 15.5]	16.4 [10.9; 23.9]
- Rural	3456 (89.9)	43.2 [37.7; 48.9]	8.4 [6.3; 11.0]	22.5 [19.0; 26.6]
- Total	3911 (100)	42.7 [37.3; 48.3]	8.2 [6.2; 10.6]	21.9 [18.5; 25.8]
**Highest level of education completed**		*P* = 0.0137	*P* = 0.3850	*P* = 0.1107
- None	1051 (24.2)	48.2 [42.0; 54.4]	9.0 [6.1; 13.1]	26.0 [21.2; 31.5]
- Some primary	1394 (34.8)	46.0 [39.2; 52.9]	9.3 [6.5; 13.1]	23.6 [19.6; 28.3]
- Completed primary	1309 (37.3)	37.2 [30.1; 44.9]	6.2 [3.8; 10.0]	17.6 [12.9; 23.6]
- Secondary and higher	144 (3.4)	31.9 [21.5; 44.5]	12.4 [5.7; 24.9]	23.3 [11.7; 41.0]
- Missing	13 (0.2)	16.9 [3.1; 56.3]	0	0
- Total	3911 (100)	42.7 [37.3; 48.3]	8.2 [6.2; 10.6]	21.9 [18.5; 25.8]
**Mother’s working status**		*P* = 0.1457	*P* = 0.9276	*P* = 0.1697
- Not worked in last 12 months	508 (15.7)	43.0 [36.0; 50.2]	7.6 [4.2; 13.2]	20.4 [13.8; 29.0]
- Not currently working but worked in last 12 months	94 (3.8)	58.5 [43.9; 71.9]	8.4 [3.7; 18.3]	37.06 [23.8; 52.7]
- Currently working	3301 (80.5)	41.9 [35.7; 48.4]	8.3 [6.3; 10.8]	21.52 [17.3; 26.5]
- Missing	8 (0.04)	30.5 [7.5; 70.4]	0	2.52 [0.3; 19.6]
- Total	3911 (100)	42.7 [37.3; 48.3]	8.2 [6.2; 10.6]	21.92 [18.5; 25.8]
**Wealth index**		*P* = 0.1298	*P* = 0.1478	*P* = 0.0032
- Low	451 (21)	50.7 [42.6; 58.7]	11.84 [7.8; 18.0]	30.2 [23.9; 37.3]
- Low middle	862 (19.5)	44.2 [38.3; 50.3]	9.12 [6.5; 12.7]	26.2 [22.0; 31.0]
- Middle	815 (20.4)	37.7 [30.8; 45.1]	6.89 [3.5; 13.3]	18.9 [14.2; 24.7]
- High middle	793 (21.5)	41.8 [30.6; 54.0]	4.97 [2.6; 9.2]	16.1 [10.2; 24.7]
- High	940 (17.7)	38.5 [30.9; 46.7]	8.07 [4.9; 12.9]	17.9 [13.5; 23.3]
- Total	3861 (100)	42.7 [37.3; 48.3]	8.16 [6.2; 10.6]	21.9 [18.5; 25.8]
***Intermediate factors***				
**Mother’s age**		*P* = 0.0885	*P* = 0.5562	*P* = 0.0437
- 15–24	1145 (28.9)	42.0 [34.9; 49.4]	8.2 [5.9; 11.3]	20.8 [16.5; 25.8]
- 25–34	1951 (51.3)	44.5 [38.6; 50.4]	8.0 [5.6; 11.2]	21.58 [17.7; 26.0]
- 35–44	748 (18)	41.4 [33.4; 49.9]	9.2 [5.8; 14.4]	26.33 [20.1; 33.7]
- 45–49	67 (1.8)	17.5 [7.9; 34.5]	2.7 [0.8; 8.7]	5.38 [1.9; 14.6]
- Total	3911 (100)	42.7 [37.3; 48.3]	8.2 [6.2; 10.6]	21.92 [18.5; 25.8]
**Marital status**		*P* = 0.2236	*P* = 0.5243	*P* = 0.2078
- Never married	59 (1.1)	42.7 [24.0; 63.8]	16.0 [5.5; 38.6]	21.5 [10.4; 39.3]
- Married/in a union	3636 (94.9)	43.1 [37.7; 48.7]	8.1 [6.1; 10.7]	22.2 [18.7; 26.1]
- Divorced/separated/widowed	216 (4)	32.8 [21.6; 46.2]	6.8 [2.1; 19.8]	15.2 [9.6; 23.3]
- Total	3911 (100)	42.7 [37.3; 48.3]	8.2 [6.2; 10.6]	21.9 [18.5; 25.8]
**Number of antenatal visits during most recent pregnancy** (***n*** **= 2394)**		*P* = 0.2964	*P* = 0.3890	*P* = 0.1657
- None	519 (29.7)	36.7 [25.9; 49.0]	9.1 [4.8; 16.6]	21.7 [14.8; 30.6]
- 1–3	905 (33)	37.2 [31.4; 43.3]	11.0 [8.0; 15.1]	21.6 [17.5; 26.2]
- 4 +	957 (35.6)	30.0 [24.3; 36.3]	8.7 [5.7; 13.2]	16.2 [11.9; 21.6]
- Don’t know	48 (1.6)	46.8 [19.5; 76.1]	3.2 [0.9; 10.6]	12.6 [4.6; 30.3]
- Missing	2 (0.04)	100	64.1 [9.8; 96.7]	100
- Total	2394 (100)	34.6 [29.0; 40.8]	9.5 [6.9; 13.0]	19.6 [15.9; 23.8]
**Mother’s age at birth**		*P* = 0.0236	*P* = 0.3507	*P* = 0.9401
- Less than 20	547 (15)	45.4 [37.5; 53.7]	9.0 [5.7; 14.0]	22.5 [17.6; 28.2]
- 20–34	2755 (69.2)	44.1 [39.1; 49.3]	7.4 [5.4; 10.1]	21.7 [18.1; 25.8]
- 35–49	607 (15.8)	33.9 [25.0; 44.0]	10.4 [6.3; 16.7]	22.5 [15.9; 30.9]
- Total	3909 (100)	42.7 [37.3; 48.3]	8.1 [6.2; 10.6]	21.9 [18.5; 25.8]
**Birth interval**		*P* = 0.1874	*P* = 0.8829	*P* = 0.0948
- First birth	706 (18.2)	42.8 [35.4; 50.4]	8.2 [5.3; 12.5]	20.0 [15.2; 25.9]
- 0–23 months	884 (22.6)	48.7 [42.5; 54.9]	8.1 [5.0; 12.9]	26.7 [21.9; 32.1]
- 24–47 months	1836 (47.6)	41.2 [33.9; 48.9]	8.5 [6.1; 11.5]	21.7 [16.7; 27.3]
- 48 + months	483 (11.6)	37.2 [27.4; 48.1]	6.8 [4.1; 10.9]	16.6 [11.3; 23.8]
- Total	3909 (100)	42.7 [37.3; 48.3]	8.1 [6.2; 10.6]	21.9 [18.5; 25.8]
**Number of living children in the household**		*P* = 0.3886	*P* = 0.8745	*P* = 0.2218
- 1–2	1216 (31)	39.9 [32.9; 47.4]	8.5 [6.0; 11.9]	20.4 [16.4; 25.2]
- 3–4	1407 (37.6)	44.2 [37.4; 51.2]	7.7 [5.3; 11.2]	20.4 [15.5; 26.]
- 5 +	1288 (31.4)	43.3 [38.0; 49.4]	8.4 [5.8; 12.0]	25.2 [20.3; 30.8]
- Total	3911 (100)	42.7 [37.3; 48.3]	8.2 [6.2; 10.6]	21.9 [18.5; 25.8]
**Place of delivery**		*P* = 0.2107	*P* = 0.6718	*P* = 0.3918
- Home	989 (33.1)	40.3 [29.6; 52.0]	9.1 [5.8; 13.9]	20.9 [14.8; 28.7]
- Health facility	2823 (64.5)	43.0 [38.7; 47.4]	7.6 [5.6; 10.3]	22.5 [19.5; 25.8]
- Missing	81 (2.4)	64.6 [45.9; 79.8]	6.2 [1.6; 21.5]	11.3 [4.5; 25.7]
- Total	3893 (100)	42.6 [37.3; 48.1]	8.1 [6.1; 10.5]	21.7 [18.3; 25.5]
**Mother had postnatal care within 42 days of delivery (*n* = 2431)**		*P* = 0.3136	*P* = 0.7939	*P* = 0.6410
- No	1475 (61.1)	34.4 [26.9; 42.7]	9.3 [6.2; 13.7]	19.4 [14.7; 25.2]
- Yes	909 (38.3)	35.5 [30.1; 41.3]	10.1 [6.8; 14.7]	20.2 [16.0; 25.1]
- Missing	10 (0.6)	6.1 [0.9; 32.7]	0	0
- Total	2494 (100)	34.6 [29.0; 40.8]	9.5 [6.9; 13.0]	19.6 [15.9; 23.8]
**Mother with no media exposure**		*P* = 0.0035	*P* = 0.8832	*P* = 0.0116
- No media exposure	2989 (81.1)	45.1 [38.8; 51.5]	8.1 [6.1; 10.7]	23.2 [19.6; 27.3]
- Some media exposure	920 (18.7)	32.2 [26.4; 38.6]	8.5 [5.5; 12.7]	15.9 [11.3; 21.9]
- Missing	2 (0.2)	50.0 [50.0; 50.0]	0	50.0 [50.0; 50.0]
- Total	3911 (100)	42.7 [37.3; 48.3]	8.2 [6.2; 10.6]	21.9 [18.5; 25.8]
**Mother listens to radio at least once a week**		*P* = 0.0762	*P* = 0.7676	*P* = 0.2713
- No	3500 (93.3)	43.2 [37.8; 48.9]	8.2 [6.2; 10.7]	22.3 [18.9; 26.2]
- Yes	383 (5.9)	35.6 [27.8; 44.2]	8.5 [4.1; 16.6]	16.3 [10.6; 24.2]
- Missing	28 (0.8)	31.8 [17.1; 51.4]	3.7 [0.5; 22.8]	17.3 [5.2; 44.4]
- Total	3911 (100)	42.7 [37.3; 48.3]	8.2 [6.2; 10.6]	21.9 [18.5; 25.8]
**Mother watches TV at least once a week**		*P* < 0.001	*P* = 0.0237	*P* = 0.0022
- No	3765 (97.6)	43.2 [37.7; 48.9]	7.9 [6.1; 10.3]	21.9 [18.5; 25.8]
- Yes	102 (1.5)	12.1 [5.9; 23.5]	10.5 [4.4; 23.1]	6.7 [2.7; 15.7]
- Missing	44 (0.9)	41.2 [25.4; 59.1]	30.4 [8.6; 66.9]	46.8 [24.8; 70.1]
- Total	3911 (100)	42.7 [37.3; 48.3]	8.2 [6.2; 10.6]	21.9 [18.5; 25.8]
**Mother reads a journal at least once a week**		*P* = 0.0493	*P* = 0.7622	*P* = 0.0048
- No	3630 (94)	43.4 [37.8; 49.2]	8.3 [6.3; 10.9]	22.3 [18.9; 26.3]
- Yes	277 (5.8)	30.7 [21.0; 42.5]	6.5 [3.2; 12.5]	14.1 [9.3; 20.8]
- Missing	4 (0.2)	50.0 [50.0; 50.0]	0	50.0 [50.0; 50.0]
- Total	3911 (100)	42.7 [37.3; 48.3]	8.2 [6.2; 10.6]	21.9 [18.5; 25.8]
**Water source**		*P* = 0.5615	*P* = 0.7452	*P* = 0.1696
- Improved	1310 (23.3)	43.2 [36.5; 50.2]	9.1 [6.72; 12.3]	22.8 [18.7; 27.5]
- Not improved	2600 (76.6)	42.5 [35.9; 49.3]	7.9 [5.68; 10.8]	21.5 [17.4; 26.4]
- Missing	1 (0.1)	100	0	100
- Total	3911 (100)	42.7 [37.3; 48.3]	8.2 [6.2; 10.6]	21.9 [18.5; 25.8]
**Toilet**		*P* = 0.1222	*P* = 0.8387	*P* = 0.0253
- Improved/not shared	283 (3.5)	35.3 [28.9; 42.4]	9.9 [3.4; 25.5]	12.9 [7.3; 21.9]
- Not improved/shared	3624 (96.2)	42.7 [37.2; 48.5]	8.1 [6.2; 10.6]	22.1 [18.6; 26.1]
- Missing	4 (0.4)	100	0	51.2 [19.6; 81.8]
- Total	3911 (100)	42.7 [37.3; 48.3]	8.2 [6.2; 10.6]	21.9 [18.5; 25.8]
***Immediate factors***				
**Birth order**		*P* = 0.7835	*P* = 0.2834	*P* = 0.1203
- 1	712 (18.4)	43.4 [36.1; 51.1]	8.5 [5.5; 12.9]	20.3 [15.5; 26.1]
- 2–3	1340 (33.6)	44.0 [36.9; 51.4]	7.4 [5.0; 10.7]	21.2 [16.6; 26.6]
- 4–5	1016 (27.1)	40.2 [31.4; 49.7]	6.9 [4.5; 10.3]	19.4 [14.2; 26.0]
- 6 +	843 (20.9)	43.1 [37.8; 48.5]	10.8 [7.0; 16.5]	27.7 [22.3; 33.8]
- Total	3911 (100)	42.7 [37.3; 48.3]	8.2 [6.2; 10.6]	21.9 [18.5; 25.8]
**Perceived size of baby at birth**		*P* = 0.0128	*P* = 0.9378	*P* = 0.2616
- Very small	54 (1.2)	65.9 [39.1; 85.3]	3.9 [1.1; 13.0]	36.3 [11.9; 70.7]
- Small	188 (4.5)	49.3 [36.7; 61.9]	8.6 [4.7; 15.3]	31.3 [20.1; 45.1]
- Average +	3510 (90.1)	41.3 [35.5; 47.4]	8.1 [6.1; 10.7]	21.0 [17.5; 24.9]
- Don’t know	72 (1.9)	37.7 [20.1; 59.3]	7.5 [1.9; 25.0]	30.1 [14.6; 52.0]
- Missing	71 (2.4)	71.0 [52.3; 84.5]	8.5 [2.6; 24.5]	17.3 [7.5; 35.0]
- Total	3895 (100)	42.6 [37.3; 48.1]	8.1 [6.2; 10.6]	21.7 [18.3; 25.5]
**Child had diarrhea in the last 2 weeks (*n* = 3876)**		*P* = 0.5148	*P* = 0.1027	*P* = 0.1377
- No	3311 (83.2)	43.1 [37.4; 49.0]	7.3 [5.4; 9.8]	20.7 [17.4; 24.4]
- Yes	518 (15.7)	39.7 [30.8; 49.5]	11.9 [7.3; 18.8]	26.7 [19.1; 36.0]
- Missing	47 (1.1)	54.0 [32.0; 74.6]	13.4 [3.5; 39.9]	27.7 [13.6; 48.2]
- Total	3876 (100)	42.7 [37.3; 48.3]	8.1 [6.2; 10.6]	21.7 [18.3; 25.6]
**Breastfeeding status of youngest children under 23 months of age (*n* = 1612)**		*P* = 0.3714	*P* = 0.4483	*P* = 0.0632
- Not breastfeeding	171 (7.6)	35.3 [24.5; 47.8]	7.3 [3.3; 15.4]	14.9 [8.3; 25.1]
- Exclusively breastfeeding	292 (15.7)	19.4 [12.5; 28.8]	6.5 [2.9; 13.9]	5.0 [2.0; 12.0]
- Breastfeeding + water	150 (13.4)	28.2 [14.6; 47.5]	10.5 [3.8; 25.7]	14.9 [8.1; 25.9]
- Breastfeeding + supplement^a^	965 (61.7)	31.1 [23.9; 39.2]	11.6 [7.8; 16.9]	17.5 [12.8; 23.4]
- Missing	34 (1.7)	28.6 [9.8; 59.6]	20.2 [5.3; 53.3]	22.6 [6.3; 56.1]
- Total	1612 (100)	29.1 [23.7; 35.2]	10.5 [7.2; 15.0]	15.1 [11.9; 18.9]

Frequencies are unweighted, percentages are weighted

^a^Breastfeeding + supplements included breastfeeding + nonmilk liquids, breastfeeding + milk and breastfeeding + foods

### Prevalence of stunting, wasting and underweight in children under five in DRC, ASSP 2014

Stunting was the most frequent nutritional impairment observed in this study (42.7%), followed by underweight (21.9%) and wasting (8.2%) (Table 2).

**Table 2:** Prevalence of stunting, wasting and underweight among children aged 0–59 months in the Democratic Republic of Congo, ASSP 2014 (*n* = 3911)

**Parameter**	**n (%) [95% CI]**
Stunting	
-No	2268 (57.3) [51.8; 62.7]
-Yes	1643 (42.7) [37.3; 48.3]
Wasting	
-No	3593 (91.8) [89.4; 93.8]
-Yes	318 (8.2) [6.2; 10.6]
Underweight	
-No	3048 (78.1) [74.3; 81.5]
-Yes	863 (21.9) [18.5; 25.8]

Frequencies are unweighted, percentages are weighted

### Prevalence and distribution of stunting, wasting and underweight by demographic and socio-economic characteristics of children under-five in DRC. ASSP 2014

There were significant differences in groups for the three measurements of undernutrition (stunted vs not stunted, wasted vs not wasted and underweight vs not underweight) by children’s age categories (all *p*-values < 0.05). The highest prevalence of stunting, underweight and wasting was found in children aged 36–47 months (55.9%), followed by 24–35 months (29.4%), and 6–11 months (17%) respectively. The province of Kasai occidental had the highest prevalence of stunting (48.5%, *p* < 0.05) and underweight (26.3%, *p* < 0.05). Children that were perceived to be born as very small were more prone to be stunted (65.9%, *p*-value = 0.0128). Children born to mothers without education (48.2%, *p* = 0.0137) were the most at risk of stunting. When comparing by wealth, children from the lowest wealth quintile were the most prone to be underweight (30.2%, *p*-value = 0.0032) (Table 1).

### Risk factors associated with stunting, wasting, and underweight among children under five years in DRC, ASSP 2014

#### Risk factors associated with stunting among children aged 0–23 months in DRC, ASSP 2014

Risk factors associated with stunting among children aged 0–23 months are presented in Table 3. The child’s age, province, education level of the mother, mother’s working status and child’s birth size were significant risk factors of stunting after controlling for other factors. Compared to children aged 0–5 months, children aged 12–23 months were more likely to be stunted (aOR = 6.53, 95% CI: 3.17; 13.44).

**Table 3:** Factors associated with stunting among children aged 0–23 months in DRC, ASSP 2014

Characteristics	Model 1			Model 2			Model 3		
	aOR	**95% CI**	***P* value**	**aOR**	**95% CI**	***P* value**	**aOR**	**95% CI**	***P* value**
**Child age in months**									
0–5	1.00			1.00			1.00		
6–11	1.41	[0.77; 2.58]	0.260	1.39	[0.73; 2.62]	0.313	2.00	[0.93; 4.31]	0.077
12–23	**3.56**	**[1.86; 6.84]**	** < 0.001**	**3.84**	**[2.09; 7.02]**	** < 0.001**	**6.53**	**[3.17; 13.44]**	** < 0.001**
**Child sex**									
Male	1.00			1.00			1.00		
Female	0.94	[0.70; 1.26]	0.669	0.88	[0.65; 1.21]	0.437	0.90	[0.66; 1.23]	0.503
***Distal factors***									
**Province**									
Equateur	1.00			1.00			1.00		
Kasai occidental	**1.97**	**[1.26; 3.07]**	**0.003**	**2.09**	**[1.24; 3.50]**	**0.005**	**2.17**	**[1.28; 3.69]**	**0.004**
Maniema/Orientale	0.77	[0.36; 1.62]	0.487	0.78	[0.39; 1.53]	0.467	0.83	[0.44; 1.59]	0.578
**Residence**									
Urban	1.00			1.00			1.00		
Rural	0.89	[0.50; 1.61]	0.709	1.33	[0.73; 2.42]	0.357	1.26	[0.64; 2.47]	0.501
**Highest level educ**									
Secondary and higher	1.00			1.00			1.00		
None	2.83	[0.56; 14.26]	0.205	**7.73**	**[1.21; 49.25]**	**0.031**	**11.90**	**[1.56; 90.92]**	**0.017**
Some primary	3.62	[0.77; 16.96]	0.102	**8.58**	**[1.50; 49.08]**	**0.016**	**13.95**	**[2.07; 94.20]**	**0.007**
Completed primary	2.73	[0.65; 11.36]	0.167	**5.44**	**[1.14; 25.92]**	**0.034**	**7.96**	**[1.37; 46.23]**	**0.021**
**Working status**									
Not worked in last 12 months	1.00			1.00			1.00		
Not currently working but worked in last 12 months	**2.80**	**[1.05; 7.52]**	**0.041**	**4.07**	**[1.38; 12.02]**	**0.011**	**4.56**	**[1.62; 12.85]**	**0.004**
Currently working	1.13	[0.64; 2.01]	0.664	1.46	[0.70; 3.06]	0.314	1.62	[0.75; 3.54]	0.221
**Wealth quintile**									
High	1.00			1.00			1.00		
High middle	1.15	[0.60; 2.22]	0.675	1.24	[0.72; 2.13]	0.444	1.12	[0.63; 1.98]	0.702
Middle	0.80	[0.42; 1.53]	0.501	0.91	[0.43; 1.91]	0.789	0.86	[0.40; 1.82]	0.686
Low middle	1.06	[0.61; 1.82]	0.842	1.24	[0.69; 2.22]	0.478	1.25	[0.66; 2.35]	0.494
Low	1.19	[0.60; 2.38]	0.611	1.42	[0.77; 2.59]	0.260	1.54	[0.77; 3.09]	0.223
***Intermediate factors***									
**Mother’s age**									
15–24 years				1.00			1.00		
25–34 years				0.55	[0.21; 1.49]	0.238	0.56	[0.20; 1.57]	0.269
35–44 years				0.83	[0.17; 4.06]	0.814	0.85	[0.15; 4.87]	0.856
45–49 years				0.48	[0.02; 9.65]	0.628	0.56	[0.02; 13.37]	0.717
**Marital status**									
Married/in a union				1.00			1.00		
Never married				2.31	[0.71; 7.46]	0.163	1.92	[0.55; 6.72]	0.309
Divorced/separated/widowed				1.65	[0.57; 4.76]	0.356	1.66	[0.59; 4.72]	0.339
**Number of antenatal visits during most recent pregnancy**									
None				1.00			1.00		
1–3				1.17	[0.66; 2.08]	0.581	1.12	[0.61; 2.05]	0.705
4 +				0.73	[0.44; 1.19]	0.203	0.71	[0.43; 1.17]	0.182
Don’t know				**4.82**	**[1.11; 20.96]**	**0.036**	4.31	[0.99; 18.87]	0.052
**Mother’s age at birth**									
20–34 years				1.00			1.00		
Less than 20 years				0.83	[0.36; 1.93]	0.666	0.90	[0.37; 2.19]	0.816
35–49 years				0.91	[0.20; 4.11]	0.905	0.81	[0.16; 4.04]	0.799
**Birth interval**									
First birth				1.00			1.00		
0–23 months				1.17	[0.54; 2.57]	0.689	0.06	[0.002; 2.02]	0.116
24–47 months				0.69	[0.30; 1.62]	0.398	0.04	[0.001; 1.28]	0.068
48 + months				0.64	[0.22; 1.88]	0.417	0.04	[0.001; 1.43]	0.077
**Number of living children in the household**									
1–2				1.00			1.00		
3–4				1.12	[0.42; 3.00]	0.825	1.27	[0.47; 3.45]	0.633
5 +				1.48	[0.53; 4.13]	0.449	1.55	[0.45; 5.33]	0.489
**Place of delivery**									
Home				1.00			1.00		
Health facility				1.55	[0.88; 2.75]	0.129	1.52	[0.83; 2.76]	0.173
**Had postnatal care within 42 days of delivery**									
No				1.00			1.00		
Yes				0.83	[0.50; 1.36]	0.451	0.84	[0.49; 1.44]	0.533
**Mother with no media exposure**									
No media exposure				1.00			1.00		
Some media exposure				1.42	[0.53; 3.80]	0.488	1.79	[0.64; 5.00]	0.264
**Mother listens to radio at least once a week**									
No				1.00			1.00		
Yes				0.57	[0.18; 1.78]	0.332	0.49	[0.14; 1.67]	0.253
**Mother watches TV at least once a week**									
No				1.00			1.00		
Yes				0.89	[0.31; 2.58]	0.831	0.68	[0.24; 1.92]	0.461
**Mother reads a journal at least once a week**									
No				1.00			1.00		
Yes				0.57	[0.22; 1.43]	0.229	0.62	[0.23; 1.70]	0.351
**Water source**									
Improved				1.00			1.00		
Not improved				1.06	[0.60; 1.87]	0.840	1.02	[0.58; 1.78]	0.950
**Toilet**									
Improved/not shared				1.00			1.00		
Not improved/shared				1.005	[0.30; 3.36]	0.993	0.97	[0.29; 3.31]	0.967
***Immediate factors***									
**Birth order**									
1							1.00		
2–3							17.43	[0.52; 579.48]	0.109
4–5							14.30	[0.40; 510.70]	0.144
6 +							17.96	[0.43; 743.55]	0.128
**Birth size**									
Average							1.00		
Very small							**12.63**	**[3.83; 41.64]**	** < 0.001**
Small							1.21	[0.44; 3.34]	0.705
Don’t know							0.58	[0.14; 2.41]	0.452
**Diarrhea in last 2 weeks**									
No							1.00		
Yes							0.96	[0.56; 1.66]	0.880
**Breastfeeding youngest under 2 years**									
Exclusively breastfeeding							1.00		
Not breastfeeding							0.53	[0.20; 1.40]	0.199
Breastfeeding + water							1.06	[0.36; 3.19]	0.910
Breastfeeding + supplement							0.56	[0.23; 1.34]	0.192
**Goodness of fit results**		F = 12.895	< 0.001		F = 4.721	< 0.001		F = 0.879	0.545

Model 1: Adjusted for child’s age, sex, and distal factors (province, residence, mother’s education, mother’s working status and wealth quintile)

Model 2: In addition to model 1, adjusted intermediate factors (mother’s age, marital status, number of antenatal visits, mother’s age at birth, birth interval, number of living children, place of delivery, postnatal care, mother’s media exposure, mother listens to radio, mother watches TV, mother reads journal, water source, toilet)

Model 3: In addition to model 2, adjusted for immediate factors (birth order, perceived birth size, diarrhea within 2 past weeks, Breastfeeding status of youngest under 2 years)

*OR* Odds ratio, *aOR* Adjusted odds ratio, *CI* Confidence interval

At the distal level, living in the province of Kasai occidental was associated with stunting, compared to living in the province of Equateur (aOR = 2.17, 95% CI: 1.28; 3.69). Stunting was more prevalent among children aged 0–23 months who had mothers with no education (aOR = 11.90, 95% CI: 1.56; 90.92), mothers with some level of primary education (aOR = 13.95, 95% CI: 2.07; 94.20) and mothers who completed primary education (aOR: 7.96, 95% CI: 1.37; 46.23) than in children who had mothers with level of secondary education and higher. Children who had mothers who did not work during the survey but worked in the last 12 months were more likely to be stunted than those who had mothers who did not work in the last 12 months (aOR = 4.56, 95% CI: 1.62; 12.85).

At the intermediate level, children who had mothers who did not know the number of antenatal visits during their latest pregnancy were more at risk of stunting compared to children with mothers who did not have attended antenatal visits. However, this association was only significant in model 2.

At the immediate level, children who were perceived to be born very small by their mothers were more likely to be stunted than those perceived to be born normal (aOR = 12.63, 95% CI: 3.83; 41.64).

#### Risk factors associated with stunting among children aged 0–59 months in DRC, ASSP 2014

Child’s age categories ranging from 12–23 months to 48–59 months had significantly higher risks of stunting compared to the 0–5 months age category and their adjusted OR remained significant in models 1, 2 and 3. Girls were less at risk of stunting than boys (aOR = 0.82, 95% CI: 0.68; 0.99) but the significance of this association was lost after including intermediate factors and immediate factors in the models. Regarding distal factors, living in the province of Kasai occidental had a positive association with stunting but also lost significance in models 2 and 3, after adjustment for intermediate and immediate factors. In the final model, children who had mothers with no education (aOR = 3.73, 95% CI: 1.06; 13.15) and some level of primary education (aOR = 3.19, 95% CI: 1.03; 9.87) were significantly more likely to be stunted compared to children who had mothers with secondary and higher education. Children who had mothers who worked in the last 12 months were more likely to be stunted than those who had mothers who did not work in the last 12 months (aOR = 3.32, 95% CI: 1.14; 9.69). Concerning intermediate factors, children born in a health facility had higher risk of stunting compared to children born at home (aOR = 1.79, 95% CI: 1.06; 3.00). At the immediate level, children perceived as very small at birth were more likely to be stunted than those perceived normal at birth (aOR = 7.87, 95% CI: 2.38; 25.98) (Table 4).

**Table 4:** Factors associated with stunting among children aged 0–59 months, ASSP 2014

Characteristics	Model 1			Model 2			Model 3		
	**aOR**	**95% CI**	***P* value**	**aOR**	**95% CI**	***P* value**	**aOR**	**95% CI**	***P* value**
**Child’s age in months**									
0–5	1.00			1.00			1.00		
6–11	1.33	[0.76; 2.32]	0.319	1.30	[0.70; 2.45]	0.407	1.31	[0.70; 2.45]	0.391
12–23	**3.25**	**[1.75; 6.06]**	** < 0.001**	**3.64**	**[1.97; 6.72]**	** < 0.001**	**3.84**	**[2.08; 7.07]**	** < 0.001**
24–35	**4.51**	**[2.64; 7.71]**	** < 0.001**	**4.19**	**[2.33; 7.51]**	** < 0.001**	**4.46**	**[2.48; 8.01]**	** < 0.001**
36–47	**5.92**	**[3.40; 10.29]**	** < 0.001**	**5.96**	**[2.83; 12.59]**	** < 0.001**	**6.29**	**[3.01; 13.10]**	** < 0.001**
48–59	**5.50**	**[3.14; 9.64]**	** < 0.001**	**9.61**	**[4.28; 21.55]**	** < 0.001**	**9.95**	**[4.37; 22.67]**	** < 0.001**
**Child sex**									
Male	1.00			1.00			1.00		
Female	**0.82**	**[0.68; 0.99]**	**0.049**	0.84	[0.67; 1.05]	0.119	0.83	[0.65; 1.06]	0.131
***Distal factors***									
**Province**									
Equateur	1.00			1.00			1.00		
Kasai occidental	**1.52**	**[1.08; 2.15]**	**0.017**	1.29	[0.79; 2.12]	0.306	1.34	[0.81; 2.21]	0.246
Maniema/Orientale	0.74	[0.42; 1.30]	0.288	0.66	[0.34; 1.27]	0.210	0.68	[0.36; 1.31]	0.249
**Residence**									
Urban	1.00			1.00			1.00		
Rural	1.05	[0.62; 1.76]	0.857	1.23	[0.65; 2.35]	0.521	1.25	[0.61; 2.53]	0.538
**Highest level educ**									
Secondary and higher	1.00			1.00			1.00		
None	1.73	[0.83; 3.62]	0.144	3.13	[0.98; 10.04]	0.055	**3.73**	**[1.06; 13.15]**	**0.040**
Some primary	1.70	[0.86; 3.36]	0.126	2.67	[0.92; 7.72]	0.070	**3.19**	**[1.03; 9.87]**	**0.045**
Completed primary	1.17	[0.64; 2.14]	0.603	2.02	[0.71; 5.71]	0.185	2.38	[0.77; 7.35]	0.132
**Working status**									
Not worked in last 12 months	1.00			1.00			1.00		
Not currently working but worked in last 12 months	1.71	[0.90; 3.25]	0.099	**3.40**	**[1.17; 9.91]**	**0.025**	**3.32**	**[1.14; 9.69]**	**0.028**
Currently working	0.93	[0.61; 1.41]	0.736	1.41	[0.78; 2.55]	0.248	1.43	[0.78; 2.62]	0.244
**Wealth quintile**									
High	1.00			1.00			1.00		
High middle	1.24	[0.75; 2.03]	0.401	1.24	[0.73; 2.10]	0.433	1.21	[0.71; 2.06]	0.479
Middle	0.99	[0.58; 1.69]	0.974	0.99	[0.57; 1.72]	0.978	0.99	[0.58; 1.70]	0.972
Low middle	1.09	[0.66; 1.79]	0.743	1.37	[0.81; 2.31]	0.246	1.32	[0.78; 2.24]	0.295
Low	1.27	[0.73; 2.22]	0.394	1.47	[0.86; 2.51]	0.158	1.48	[0.87; 2.52]	0.145
***Intermediate factors***									
**Mother’s age**									
15–24 years				1.00			1.00		
25–34 years				0.87	[0.43; 1.77]	0.703	0.85	[0.41; 1.75]	0.658
35–44 years				2.28	[0.78; 6.61]	0.130	2.07	[0.73; 5.90]	0.172
45–49 years				0.38	[0.07; 2.18]	0.276	0.36	[0.06; 2.19]	0.264
**Marital status**									
Married/in a union				1.00			1.00		
Never married				**2.87**	**[1.15; 7.21]**	**0.025**	2.71	[0.98; 7.51]	0.055
Divorced/separated/widowed				0.97	[0.53; 1.78]	0.934	1.04	[0.56; 1.91]	0.901
**Number of antenatal visits during most recent pregnancy**									
None				1.00			1.00		
1–3				1.06	[0.67; 1.68]	0.792	1.03	[0.65; 1.63]	0.892
4 +				0.65	[0.39; 1.08]	0.096	0.66	[0.40; 1.08]	0.096
Don’t know				1.00	[0.26; 3.92]	1.000	1.04	[0.25; 4.33]	0.960
**Mother’s age at child’s birth**									
20–34 years				1.00			1.00		
Less than 20 years				1.23	[0.63; 2.43]	0.542	1.28	[0.63; 2.58]	0.491
35–49 years				0.42	[0.17; 1.02]	0.054	0.43	[0.17; 1.05]	0.064
**Birth interval**									
First birth				1.00			1.00		
0–23 months				1.25	[0.66; 2.39]	0.496	1.74	[0.16; 18.71]	0.645
24–47 months				0.66	[0.33; 1.35]	0.256	0.94	[0.09; 10.08]	0.961
48 + months				0.52	[0.24; 1.15]	0.108	0.74	[0.07; 7.44]	0.800
**Number of living children in the household**									
1–2				1.00			1.00		
3–4				1.19	[0.66; 2.15]	0.557	1.17	[0.60; 2.27]	0.648
5 +				1.13	[0.58; 2.17]	0.723	1.00	[0.46; 2.21]	0.989
**Place of delivery**									
Home				1.00			1.00		
Health facility				**1.79**	**[1.07; 2.99]**	**0.027**	**1.79**	**[1.06; 3.00]**	**0.029**
**Had postnatal care within 42 days of delivery**									
No				1.00			1.00		
Yes				0.90	[0.64; 1.27]	0.547	0.88	[0.62; 1.25]	0.483
**Mother with no media exposure**									
No media exposure				1.00			1.00		
Some media exposure				1.21	[0.63; 2.35]	0.566	1.22	[0.62; 2.40]	0.567
**Mother listens to radio at least once a week**									
No				1.00			1.00		
Yes				0.56	[0.27; 1.15]	0.114	0.59	[0.29; 1.21]	0.147
**Mother watches TV at least once a week**									
No				1.00			1.00		
Yes				0.66	[0.23; 1.94]	0.452	0.59	[0.20; 1.73]	0.339
**Mother reads a journal at least once a week**									
No				1.00			1.00		
Yes				0.59	[0.24; 1.48]	0.260	0.62	[0.24; 1.55]	0.304
**Water source**									
Improved				1.00			1.00		
Not improved				1.00	[0.65; 1.56]	0.984	1.03	[0.65; 1.61]	0.914
**Toilet**									
Improved/not shared				1.00			1.00		
Not improved/shared				0.98	[0.39; 2.46]	0.964	0.95	[0.37; 2.47]	0.921
***Immediate factors***									
**Birth order**									
1							1.00		
2–3							0.72	[0.07; 7.07]	0.774
4–5							0.74	[0.07; 7.25]	0.792
6 +							0.84	[0.08; 9.01]	0.882
**Birth size**									
Average							1.00		
Very small							**7.87**	**[2.38; 25.98]**	**0.001**
Small							1.59	[0.85; 2.94]	0.143
Don’t know							0.89	[0.29; 2.71]	0.836
**Diarrhea in last 2 weeks**									
No							1.00		
Yes							0.94	[0.58; 1.50]	0.783
**Result of Goodness of fit test**		F = 3.333	0.001		F = 1.000	0.441		F = 1.191	0.301

Model 1: Adjusted for child’s age, sex, and distal factors (province, residence, mother’s education, mother’s working status and wealth quintile)

Model 2: In addition to model 1, adjusted intermediate factors (mother’s age, marital status, number of antenatal visits, mother’s age at birth, birth interval, number of living children, place of delivery, postnatal care, mother’s media exposure, mother listens to radio, mother watches TV, mother reads journal, water source, toilet)

Model 3: In addition to model 2, adjusted for immediate factors (birth order, perceived birth size, diarrhea within 2 past weeks)

*OR* Odds ratio, *aOR* Adjusted odds ratio, *CI* Confidence interval

#### Risk factors associated wasting among children aged 0–23 months in DRC, ASSP 2014

At the distal level, children who resided in rural areas were more likely to be wasted (aOR = 3.42, 95% CI: 1.14; 10.30) in model 1 but the significance of this association was lost in subsequent models. No significant association was found for intermediate factors. At the immediate factors level, children who were breastfed in addition to drinking water were more likely to be wasted compared to children exclusively breastfed and the association was significant (aOR = 2.56; 95% CI: 1.01; 6.47) (Table 5).

**Table 5:** Factors associated with wasting aged 0–23 months in DRC, ASSP 2014

Characteristics	Model 1			Model 2			Model 3		
	**aOR**	**95% CI**	***P* value**	**aOR**	**95% CI**	***P* value**	**aOR**	**95% CI**	***P* value**
**Child’s age in months**									
0–5	1.00			1.00			1.00		
6–11	1.95	[0.99; 3.87]	0.055	1.74	[0.91; 3,34]	0.095	1.12	[0.51; 2.50]	0.774
12–23	0.99	[0.48; 2.07]	0.998	0.76	[0.36; 1.59]	0.465	0.47	[0.19; 1.18]	0.108
**Child sex**									
Male	1.00			1.00			1.00		
Female	0.62	[0.32; 1.20]	0.153	0.69	[0.33; 1.42]	0.311	0.68	[0.33; 1.40]	0.294
***Distal factors***									
**Province**									
Equateur	1.00			1.00			1.00		
Kasai occidental	1.00	[0.53; 1.89]	1.000	1.11	[0.48; 2.56]	0.800	0.70	[0.28; 1.76]	0.442
Maniema/Orientale	1.96	[0.81; 4.75]	0.134	2.02	[0.76; 5.33]	0.157	1.77	[0.65; 4.77]	0.260
**Residence**									
Urban	1.00			1.00			1.00		
Rural	**3.42**	**[1.14; 10.30]**	**0.029**	2.83	[0.97; 8.24]	0.056	3.43	[0.99; 11.82]	0.051
**Highest level educ**									
Secondary and higher	1.00			1.00			1.00		
None	0.48	[0.13; 1.76]	0.265	0.56	[0.10; 3.20]	0.515	0.65	[0.11; 3.87]	0.638
Some primary	0.48	[0.14; 1.68]	0.252	0.52	[0.11; 2.55]	0.420	0.56	[0.11; 2.85]	0.480
Completed primary	0.36	[0.10; 1.27]	0.112	0.40	[0.09; 1.86]	0.242	0.41	[0.09; 1.88]	0.247
**Working status**									
Not worked in last 12 months	1.00			1.00			1.00		
Not currently working but worked in last 12 months	1.21	[0.27; 5.48]	0.804	1.25	[0.31; 5.10]	0.751	0.81	[0.16; 4.06]	0.797
Currently working	0.88	[0.51; 1.51]	0.638	0.82	[0.46; 1.47]	0.500	0.64	[0.36; 1.12]	0.114
**Wealth quintile**									
High	1.00			1.00			1.00		
High middle	0.48	[0.19; 1.23]	0.128	0.60	[0.21; 1.66]	0.322	0.50	[0.17; 1.44]	0.195
Middle	0.92	[0.35; 2.42]	0.871	1.19	[0.51; 2.75]	0.688	1.08	[0.42; 2.74]	0.876
Low middle	1.05	[0.49; 2.27]	0.903	1.28	[0.55; 2.97]	0.558	1.15	[0.51; 2.60]	0.741
Low	1.88	[0.80; 4.43]	0.148	2.38	[0.97; 5.88]	0.059	1.87	[0.74; 4.72]	0.186
***Intermediate factors***									
**Mother’s age**									
15–24 years				1.00			1.00		
25–34 years				1.45	[0.49; 4.30]	0.499	1.21	[0.37; 3.95]	0.750
35–44 years				1.47	[0.25; 8.62]	0.667	1.06	[0.15; 7.59]	0.951
45–49 years				0.64	[0.05; 9.01]	0.738	0.35	[0.02; 5.99]	0.466
**Marital status**									
Married/in a union				1.00			1.00		
Never married				1.12	[0.33; 3.88]	0.854	1.24	[0.37; 4.20]	0.724
Divorced/separated/widowed				0.55	[0.10; 3.12]	0.498	0.50	[0.08; 3.09]	0.456
**Number of antenatal visits during most recent pregnancy**									
None				1.00			1.00		
1–3				1.46	[0.60; 3.56]	0.399	1.30	[0.51; 3.32]	0.578
4 +				1.11	[0.45; 2.77]	0.819	0.98	[0.40; 2.39]	0.966
Don’t know				1.00			1.00		
**Mother’s age at child’s birth**									
20–34 years				1.00			1.00		
Less than 20 years				1.12	[0.40; 3.11]	0.826	1.05	[0.40; 2.76]	0.917
35–49 years				1.57	[0.40; 6.19]	0.520	1.52	[0.34; 6.83]	0.580
**Birth interval**									
First birth				1.00			1.00		
0–23 months				0.69	[0.25; 1.90]	0.476	1.20	[0.14; 9.92]	0.867
24–47 months				0.83	[0.32; 2.18]	0.711	1.48	[0.19; 11.24]	0.705
48 + months				0.65	[0.14; 3.10]	0.590	1.18	[0.09; 15.40]	0.900
**Number of living children in the household**									
1–2				1.00			1.00		
3–4				0.90	[0.29; 2.82]	0.853	0.95	[0.36; 2.53]	0.923
5 +				0.96	[0.33; 2.73]	0.932	0.49	[0.15; 1.67]	0.255
**Place of delivery**									
Home				1.00			1.00		
Health facility				0.73	[0.32; 1.63]	0.435	0.65	[0.28; 1.50]	0.311
**Had postnatal care within 42 days of delivery**									
No				1.00			1.00		
Yes				1.02	[0.49; 2.12]	0.965	1.12	[0.55; 2.30]	0.747
**Mother with no media exposure**									
No media exposure				1.00			1.00		
Some media exposure				1.14	[0.45; 2.85]	0.781	1.36	[0.53; 3.48]	0.520
**Mother listens to radio at least once a week**									
No				1.00			1.00		
Yes				1.52	[0.50; 4.64]	0.462	1.39	[0.38; 5.03]	0.617
**Mother watches TV at least once a week**									
No				1.00			1.00		
Yes				0.35	[0.04; 3.30]	0.359	0.29	[0.05; 1.68]	0.165
**Mother reads a journal at least once a week**									
No				1.00			1.00		
Yes				1.02	[0.35; 3.04]	0.962	0.94	[0.30; 2.97]	0.920
**Water source**									
Improved				1.00			1.00		
Not improved				0.66	[0.28; 1.57]	0.349	0.64	[0.26; 1.56]	0.323
**Toilet**									
Improved/not shared				1.00			1.00		
Not improved/shared				0.57	[0.09; 3.47]	0.537	0.50	[0.08; 3.29]	0.471
***Immediate factors***									
**Birth order**									
1							1.00		
2–3							0.56	[0.08; 3.82]	0.556
4–5							0.50	[0.08; 3.19]	0.463
6 +							1.60	[0.18; 14.03]	0.668
**Birth size**									
Average							1.00		
Very small							0.25	[0.05; 1.21]	0.085
Small							1.08	[0.31; 3.78]	0.908
Don’t know							0.68	[0.11; 4.29]	0.685
**Diarrhea in last 2 weeks**									
No							1.00		
Yes							1.47	[0.73; 2.95]	0.279
**Breastfeeding youngest under 2 years**									
Exclusively breastfeeding							1.00		
Not breastfeeding							1.28	[0.30; 5.47]	0.742
Breastfeeding + water							**2.56**	**[1.01; 6.47]**	**0.047**
Breastfeeding + supplement							2.84	[0.92; 8.82]	0.071
**Result of Goodness of fit test**		F = 2.544	0.008		F = 1.709	0.087		F = 0.687	0.721

Model 1: Adjusted for child’s age, sex, and distal factors (province, residence, mother’s education, mother’s working status and wealth quintile)

Model 2: In addition to model 1, adjusted intermediate factors (mother’s age, marital status, number of antenatal visits, mother’s age at birth, birth interval, number of living children, place of delivery, postnatal care, mother’s media exposure, mother listens to radio, mother watches TV, mother reads journal, water source, toilet)

Model 3: In addition to model 2, adjusted for immediate factors (birth order, perceived birth size, diarrhea within 2 past weeks, Breastfeeding status of youngest under 2 years)

*OR* Odds ratio, *aOR* Adjusted odds ratio, *CI* Confidence interval

#### Risk factors associated with wasting among children aged 0–59 months in DRC, ASSP 2014

Children aged 6–11 months had higher risk of wasting compared to children aged 0–5 months (aOR = 2.01, 95% CI: 1.04; 3.89) but the significance of this association was lost in the subsequent models. Among distal factors, the province was found to be significantly associated with wasting in model 1 but the significance of this association was lost in the following models. Children living in province Orientale and Maniema were more likely to be wasted compared to children living in Equateur (aOR = 1.96, 95% CI: 1.08; 3.55). At the intermediate level, mother’s age and mother’s age at child’s birth had a significant relationship with wasting. Children with mothers aged 45–49 years were less likely wasted (aOR = 0.13, 95% CI: 0.02; 0.99) but the statistical significance was lost in the following model. Children who were born to mothers aged 35–49 years had higher risk of wasting in model 2 (aOR = 5.44, 95% CI: 1.71; 17.25) and model 3 (aOR = 5.32, 95% CI: 1.67; 16.89) compared to children born to mother aged between 20 and 34 years. At the immediate factors level, we did not find a significant association with wasting (Table 6).

**Table 6:** Factors associated with wasting among children aged 0–59 months in DRC, ASSP 2014

Characteristics	Model 1			Model 2			Model 3		
	**aOR**	**95% CI**	***P* value**	**aOR**	**95% CI**	***P* value**	**aOR**	**95% CI**	***P* value**
**Child’s age in months**									
0–5	1.00			1.00			1.00		
6–11	**2.01**	**[1.04; 3.89]**	**0.038**	1.78	[0.93; 3.41]	0.081	1.61	[0.88; 2.95]	0.120
12–23	1.05	[0.49; 2.22]	0.908	0.80	[0.39; 1.67]	0.553	0.77	[0.39; 1.52]	0.453
24–35	0.89	[0.41; 1.96]	0.777	1.26	[0.54; 2.95]	0.599	1.32	[0.59; 2.94]	0.503
36–47	0.46	[0.18; 1.18]	0.107	0.53	[0.12; 2.21]	0.379	0.53	[0.12; 2.32]	0.402
48–59	0.45	[0.17; 1.23]	0.121	0.57	[0.19; 1.72]	0.317	0.56	[0.20; 1.58]	0.274
**Child sex**									
Male	1.00			1.00			1.00		
Female	0.73	[0.47; 1.14]	0.162	0.62	[0.35; 1.11]	0.105	0.60	[0.33; 1.08]	0.089
***Distal factors***									
**Province**									
Equateur	1.00			1.00			1.00		
Kasai occidental	1.02	[0.64; 1.62]	0.947	1.30	[0.65; 2.58]	0.454	1.20	[0.59; 2.48]	0.610
Maniema/Orientale	**1.96**	**[1.08; 3.55]**	**0.027**	2.15	[0.98; 4.71]	0.056	2.19	[0.99; 4.79]	0.051
**Residence**									
Urban	1.00			1.00			1.00		
Rural	1.13	[0.42; 3.04]	0.809	1.73	[0.67; 4.45]	0.253	1.72	[0.65; 4.55]	0.274
**Highest level educ**									
Secondary and higher	1.00			1.00			1.00		
None	0.73	[0.26; 2.07]	0.556	0.56	[0.15; 2.10]	0.388	0.57	[0.15; 2.13]	0.403
Some primary	0.77	[0.30; 1.94]	0.574	0.54	[0.16; 1.82]	0.317	0.52	[0.16; 1.74]	0.289
Completed primary	0.54	[0.21; 1.43]	0.218	0.41	[0.13; 1.34]	0.139	0.43	[0.13; 1.36]	0.150
**Working status**									
Not worked in last 12 months	1.00			1.00			1.00		
Not currently working but worked in last 12 months	1.05	[0.43; 2.61]	0.907	1.81	[0.63; 5.19]	0.271	1.55	[0.50; 4.86]	0.450
Currently working	1.04	[0.66; 1.65]	0.859	0.96	[0.56; 1.64]	0.871	0.90	[0.52; 1.56]	0.718
**Wealth quintile**									
High	1.00			1.00			1.00		
High middle	0.55	[0.25; 1.22]	0.140	0.67	[0.32; 1.42]	0.296	0.70	[0.34; 1.47]	0.344
Middle	0.70	[0.31; 1.59]	0.396	1.05	[0.48; 2.26]	0.909	1.07	[0.48; 2.38]	0.859
Low middle	1.11	[0.59; 2.10]	0.741	1.39	[0.69; 2.82]	0.355	1.43	[0.70; 2.90]	0.323
Low	1.74	[0.85; 3.53]	0.127	1.83	[0.86; 3.89]	0.118	1.77	[0.82; 3.80]	0.143
***Intermediate factors***									
**Mother’s age**									
15–24 years				1.00			1.00		
25–34 years				1.34	[0.55; 3.23]	0.517	1.33	[0.52; 3.40]	0.551
35–44 years				0.38	[0.09; 1.53]	0.171	0.33	[0.08; 1.28]	0.107
45–49 years				**0.13**	**[0.02; 0.99]**	**0.050**	0.11	[0.01; 0.83]	0.344
**Marital status**									
Married/in a union				1.00			1.00		
Never married				0.89	[0.20; 4.04]	0.881	0.92	[0.21; 4.04]	0.911
Divorced/separated/widowed				0.45	[0.13; 1.60]	0.217	0.46	[0.13; 1.62]	0.226
**Number of antenatal visits during most recent pregnancy**									
None				1.00			1.00		
1–3				1.43	[0.68; 2.99]	0.347	1.39	[0.65; 3.01]	0.397
4 +				1.04	[0.47; 2.31]	0.916	1.01	[0.44; 2.28]	0.989
Don’t know				0.61	[0.13; 2.79]	0.518	0.62	[0.13; 2.89]	0.544
**Mother’s age at child’s birth**									
20–34 years				1.00			1.00		
Less than 20 years				1.14	[0.51; 2.52]	0.753	1.18	[0.53; 2.64]	0.686
35–49 years				**5.44**	**[1.71; 17.25]**	**0.004**	**5.32**	**[1.67; 16.89]**	**0.005**
**Birth interval**									
First birth				1.00			1.00		
0–23 months				0.80	[0.37; 1.75]	0.580	0.54	[0.12; 2.34]	0.408
24–47 months				1.13	[0.52; 2.46]	0.758	0.78	[0.20; 3.09]	0.722
48 + months				0.54	[0.17; 1.77]	0.307	0.38	[0.07; 2.03]	0.256
**Number of living children in the household**									
1–2				1.00			1.00		
3–4				0.79	[0.37; 1.67]	0.532	0.77	[0.40; 1.50]	0.446
5 +				0.90	[0.43; 1.88]	0.783	0.60	[0.24; 1.50]	0.270
**Place of delivery**									
Home				1.00			1.00		
Health facility				0.75	[0.37; 1.54]	0.435	0.72	[0.35; 1.48]	0.364
**Had postnatal care within 42 days of delivery**									
No				1.00			1.00		
Yes				0.99	[0.55; 1.80]	0.992	0.99	[0.54; 1.80]	0.970
**Mother with no media exposure**									
No media exposure				1.00			1.00		
Some media exposure				0.93	[0.40; 2.16]	0.870	0.99	[0.43; 2.25]	0.977
**Mother listens to radio at least once a week**									
No				1.00			1.00		
Yes				1.24	[0.41; 3.75]	0.698	1.20	[0.37; 3.91]	0.758
**Mother watches TV at least once a week**									
No				1.00			1.00		
Yes				0.71	[0.13; 3.94]	0.696	0.73	[0.13; 4.20]	0.720
**Mother reads a journal at least once a week**									
No				1.00			1.00		
Yes				0.87	[0.34; 2.25]	0.774	0.82	[0.31; 2.17]	0.690
**Water source**									
Improved				1.00			1.00		
Not improved				0.70	[0.38; 1.30]	0.256	0.71	[0.38; 1.31]	0.265
**Toilet**									
Improved/not shared				1.00			1.00		
Not improved/shared				0.59	[0.18; 1.97]	0.390	0.56	[0.17; 1.92]	0.359
***Immediate factors***									
**Birth order**									
1							1.00		
2–3							1.50	[0.39; 5.77]	0.556
4–5							1.40	[0.36; 5.42]	0.622
6 +							2.90	[0.63; 13.29]	0.169
**Birth size**									
Average							1.00		
Very small							1.18	[0.24; 5.70]	0.839
Small							1.73	[0.71; 4.17]	0.225
Don’t know							0.26	[0.05; 1.46]	0.127
**Diarrhea in last 2 weeks**									
No							1.00		
Yes							1.49	[0.85; 2.64]	0.166
**Result of Goodness of fit test**		F = 2.302	0.017		F = 2.359	0.014		F = 0.957	0.476

Model 1: Adjusted for child’s age, sex, and distal factors (province, residence, mother’s education, mother’s working status and wealth quintile)

Model 2: In addition to model 1, adjusted intermediate factors (mother’s age, marital status, number of antenatal visits, mother’s age at birth, birth interval, number of living children, place of delivery, postnatal care, mother’s media exposure, mother listens to radio, mother watches TV, mother reads journal, water source, toilet)

Model 3: In addition to model 2, adjusted for immediate factors (birth order, perceived birth size, diarrhea within 2 past weeks)

*OR* Odds ratio, *aOR* Adjusted odds ratio, *CI* Confidence interval

#### Risk factors associated with underweight among children aged 0–23 months in DRC, ASSP 2014

Compared to children aged less than 6 months, children aged 12–23 months were more significantly prone to underweight in all three models, while children aged 6–11 months were at significantly higher risk of underweight in models 1 and 2 only. Province was the only distal factor associated with underweight and the Kasai occidental province was associated with significant higher risk of underweight in model 1 and 2 only. At the intermediate level, children with mothers aged 45–49 years were less at risk of underweight (aOR = 0.04, 95% CI: 0.002; 0.84) but the significance of this association was lost in the following model. At the immediate level, children perceived as very small at birth were at a significantly higher risk of underweight compared to children perceived as average (aOR = 18.36, 95% CI: 2.54; 132.58) (Table 7).

**Table 7:** Factors associated with underweight among children aged 0–23 months in DRC, ASSP 2014

Characteristics	Model 1			Model 2			Model 3		
	**aOR**	**95% CI**	***P* value**	**aOR**	**95% CI**	***P* value**	**aOR**	**95% CI**	***P* value**
**Child’s age in months**									
0–5	1.00			1.00			1.00		
6–11	**2.38**	**[1.00; 5.66]**	**0.050**	**2.59**	**[1.07; 6.28]**	**0.035**	2.05	[0.81; 5.18]	0.130
12–23	**2.86**	**[1.24; 6.56]**	**0.013**	**3.17**	**[1.38; 7.31]**	**0.007**	**2.75**	**[1.16; 6.52]**	**0.022**
**Child sex**									
Male	1.00			1.00			1.00		
Female	0.86	[0.44; 2.23]	0.516	0.88	[0.56; 1.39]	0.591	0.86	[0.55; 1.35]	0.499
***Distal factors***									
**Province**									
Equateur	1.00			1.00			1.00		
Kasai occidental	**1.68**	**[1.02; 2.77]**	**0.042**	**2.08**	**[1.14; 3.81]**	**0.018**	1.81	[0.91; 3.60]	0.092
Maniema/Oriental	1.08	[0.59; 1.99]	0.804	1.23	[0.61; 2.48]	0.563	1.35	[0.63; 2.90]	0.440
**Residence**									
Urban	1.00			1.00			1.00		
Rural	0.99	[0.44; 2.23]	0.982	1.72	[0.67; 4.47]	0.261	2.27	[0.92; 5.62]	0.077
**Highest level educ**									
Secondary and higher	1.00			1.00			1.00		
None	0.58	[0.18; 1.84]	0.353	0.65	[0.19; 2.26]	0.497	0.78	[0.20; 3.06]	0.725
Some primary	0.56	[0.16; 1.95]	0.363	0.57	[0.15; 2.10]	0.396	0.66	[0.15; 2.85]	0.579
Completed primary	0.57	[0.19; 1.73]	0.322	0.55	[0.17; 1.71]	0.298	0.56	[0.16; 1.95]	0.364
**Working status**									
Not worked in last 12 months	1.00			1.00			1.00		
Not currently working but worked in last 12 months	1.85	[0.44; 7.71]	0.397	1.36	[0.26; 7.20]	0.717	1.05	[0.21; 5.23]	0.953
Currently working	0.85	[0.35; 2.10]	0.727	0.80	[0.28; 2.25]	0.669	0.68	[0.23; 2.04]	0.495
**Wealth quintile**									
High	1.00			1.00			1.00		
High middle	0.92	[0.32; 2.70]	0.885	0.83	[0.36; 1.91]	0.656	0.55	[0.23; 1.32]	0.177
Middle	1.39	[0.63; 3.05]	0.412	1.67	[0.83; 3.34]	0.149	1.56	[0.74; 3.27]	0.242
Low middle	1.72	[0.70; 4.23]	0.238	1.64	[0.74; 3.64]	0.219	1.41	[0.63; 3.16]	0.398
Low	1.49	[0.59; 3.81]	0.399	1.42	[0.56; 3.64]	0.459	1.32	[0.52; 3.35]	0.559
***Intermediate factors***									
**Mother’s age**									
15–24 years				1.00			1.00		
25–34 years				0.47	[0.15; 1.46]	0.191	0.49	[0.14; 1.72]	0.261
35–44 years				0.78	[0.10; 6.00]	0.811	0.77	[0.08; 6.99]	0.813
45–49 years				**0.04**	**[0.002; 0.84]**	**0.038**	0.04	[0.002; 1.09]	0.056
**Marital status**									
Married/in a union				1.00			1.00		
Never married				2.60	[0.57; 11.93]	0.217	2.38	[0.44; 12.94]	0.313
Divorced/separated/widowed				1.85	[0.64; 5.36]	0.255	1.81	[0.64; 5.11]	0.260
**Number of antenatal visits during most recent pregnancy**									
None				1.00			1.00		
1–3				1.71	[0.85; 3.44]	0.135	1.61	[0.79; 3.28]	0.192
4 +				0.99	[0.45; 2.20]	0.989	0.99	[0.43; 2.27]	0.982
Don’t know				1.00			1.00		
**Mother’s age at child’s birth**									
20–34 years				1.00			1.00		
Less than 20 years				0.86	[0.38; 1.95]	0.716	0.87	[0.38; 1.99]	0.743
35–49 years				0.76	[0.14; 4.26]	0.758	0.72	[0.13; 4.15]	0.717
**Birth interval**									
First birth				1.00			1.00		
0–23 months				1.83	[0.79; 4.21]	0.155	0.76	[0.12; 4.94]	0.772
24–47 months				1.66	[0.75; 3.67]	0.214	0.82	[0.11; 6.01]	0.848
48 + months				0.71	[0.21; 2.42]	0.587	0.33	[0.03; 3.25]	0.339
**Number of living children in the household**									
1–2				1.00			1.00		
3–4				1.19	[0.47; 3.03]	0.712	1.57	[0.59; 4.17]	0.366
5 +				2.44	[0.85; 6.99]	0.096	3.70	[0.90; 15.15]	0.069
**Place of delivery**									
Home				1.00			1.00		
Health facility				0.71	[0.37; 1.35]	0.289	0.74	[0.39; 1.41]	0.356
**Had postnatal care within 42 days of delivery**									
No				1.00			1.00		
Yes				0.91	[0.47; 1.79]	0.792	0.85	[0.46; 1.59]	0.610
**Mother with no media exposure**									
No media exposure				1.00			1.00		
Some media exposure				0.91	[0.37; 1.79]	0.839	0.96	[0.38; 2.44]	0.934
**Mother listens to radio at least once a week**									
No				1.00			1.00		
Yes				1.24	[0.32; 4.78]	0.753	1.22	[0.30; 4.95]	0.783
**Mother watches TV at least once a week**									
No				1.00			1.00		
Yes				0.36	[0.07; 2.03]	0.248	0.28	[0.06; 1.31]	0.105
**Mother reads a journal at least once a week**									
No				1.00			1.00		
Yes				0.95	[0.36; 2.47]	0.911	1.11	[0.38; 3.25]	0.855
**Water source**									
Improved				1.00			1.00		
Not improved				0.64	[0.32; 1.27]	0.202	0.56	[0.28; 1.12]	0.100
**Toilet**									
Improved/not shared				1.00			1.00		
Not improved/shared				1.17	[0.27; 5.14]	0.833	1.13	[0.23; 5.47]	0.882
***Immediate factors***									
**Birth order**									
1							1.00		
2–3							2.16	[0.32; 14.63]	0.429
4–5							1.05	[0.15; 7.54]	0.958
6 +							1.45	[0.17; 12.11]	0.730
**Birth size**									
Average							1.00		
Very small							**18.36**	**[2.54; 132. 58]**	**0.004**
Small							3.06	[0.99; 9.46]	0.053
Don’t know							1.22	[0.30; 5.05]	0.780
**Diarrhea in last 2 weeks**									
No							1.00		
Yes							1.66	[0.96; 2.86]	0.067
**Breastfeeding youngest under 2 years**									
Exclusively breastfeeding							1.00		
Not breastfeeding							1.89	[0.45; 7.93]	0.383
Breastfeeding + water							2.92	[0.63; 13.52]	0.169
Breastfeeding + supplement							2.23	[0.51; 9.75]	0.286
**Results of the Goodness of fit test**		F = 6.769	< 0.001		F = 3.483	< 0.001		F = 1.558	0.128

Model 1: Adjusted for child’s age, sex, and distal factors (province, residence, mother’s education, mother’s working status and wealth quintile)

Model 2: In addition to model 1, adjusted intermediate factors (mother’s age, marital status, number of antenatal visits, mother’s age at birth, birth interval, number of living children, place of delivery, postnatal care, mother’s media exposure, mother listens to radio, mother watches TV, mother reads journal, water source, toilet)

Model 3: In addition to model 2, adjusted for immediate factors (birth order, perceived birth size, diarrhea within 2 past weeks, Breastfeeding status of youngest under 2 years). OR odds ratio, aOR adjusted odds ratio, CI confidence interval

#### Risk factors associated with underweight among children aged 0–59 months in DRC, ASSP 2014

Child’s age categories from 13–23 months to 48–59 months were significantly associated with higher risk of underweight in all the three models, compared to the child’s aged category of less than 6 months. At the distal level, region and mother’s working status were found to be associated with underweight. Living in the province of Kasai occidental presented a higher risk of underweight in all the three models compared to living in Equateur, compared to living in Equateur province. Children who had mothers who were not working during the survey but worked in the last 12 months were at higher risk of underweight compared to children who had mothers who did not work in the last 12 months. Among intermediate factors, only mother’s age was statistically associated with underweight but only in model 2 such as children whose mother aged 45–49 years had lower risk of underweight (aOR = 0.13, 95% CI: 0.02; 0.80). At immediate level, perception of birth size was statistically associated with underweight. Children perceived as very small and small were at higher risk of underweight compared to children perceived as average (aOR = 8.16, 95% CI: 1.38; 48.34, and aOR = 3.44, 95% CI: 1.49; 7.94) (Table 8).

**Table 8:** Factors associated with underweight among children aged 0–59 months in DRC, ASSP 2014

Characteristics	Model 1			Model 2			Model 3		
	**aOR**	**95% CI**	***P* value**	**aOR**	**95% CI**	***P* value**	**aOR**	**95% CI**	***P* value**
**Child’s age in months**									
0–5	1.00			1.00			1.00		
6–11	2.36	[0.98; 5.71]	0.056	2.31	[0.90; 5.91]	0.081	2.14	[0.85; 5.40]	0.106
12–23	**2.90**	**[1.25; 6.73]**	**0.014**	**3.13**	**[1.30; 7.57]**	**0.011**	**3.29**	**[1.39; 7.79]**	**0.007**
24–35	**4.57**	**[2.19; 9.54]**	** < 0.001**	**5.63**	**[2.48; 12.80]**	** < 0.001**	**6.20**	**[2.68; 14.35]**	** < 0.001**
36–47	**3.77**	**[1.62; 8.78]**	**0.002**	**4.91**	**[2.19; 11.00]**	** < 0.001**	**5.59**	**[2.48; 12.60]**	** < 0.001**
48–59	**3.81**	**[1.70; 8.53]**	** < 0.001**	**8.27**	**[3.05; 22.38]**	** < 0.001**	**8.59**	**[3.12; 23.61]**	** < 0.001**
**Child sex**									
Male	1.00			1.00			1.00		
Female	0.84	[0.66; 1.06]	0.141	0.84	[0.60; 1.17]	0.305	0.81	[0.57; 1.14]	0.226
***Distal factors***									
**Province**									
Equateur	1.00			1.00			1.00		
Kasai occidental	**1.48**	**[1.06; 2.08]**	**0.022**	**1.71**	**[1.00; 2.90]**	**0.046**	**1.79**	**[1.05; 3.05]**	**0.033**
Maniema/Oriental	0.75	[0.44; 1.27]	0.283	0.84	[0.45; 1.60]	0.601	0.88	[0.46; 1.68]	0.701
**Residence**									
Urban	1.00			1.00			1.00		
Rural	1.20	[0.71; 2.03]	0.489	1.51	[0.70; 3.29]	0.293	1.67	[0.76; 3.68]	0.203
**Highest level educ**									
Secondary and higher	1.00			1.00			1.00		
None	0.95	[0.32; 2.85]	0.934	0.90	[0.32; 2.48]	0.834	0.88	[0.31; 2.53]	0.819
Some primary	0.91	[0.29; 2.85]	0.869	0.77	[0.27; 2.19]	0.626	0.76	[0.27; 2.14]	0.597
Completed primary	0.72	[0.27; 1.96]	0.521	0.60	[0.23; 1.56]	0.297	0.60	[0.22; 1.61]	0.309
**Working status**									
Not worked in last 12 months	1.00			1.00			1.00		
Not currently working but worked in last 12 months	2.02	[0.85; 4.80]	0.112	**3.51**	**[1.13; 10.89]**	**0.030**	**3.46**	**[1.15; 10.43]**	**0.028**
Currently working	1.04	[0.59; 1.86]	0.884	1.18	[0.56; 2.49]	0.656	1.18	[0.56; 2.47]	0.667
**Wealth quintile**									
High	1.00			1.00			1.00		
High middle	0.93	[0.51; 1.68]	0.800	1.09	[0.51; 2.33]	0.814	1.04	[0.53; 2.04]	0.906
Middle	1.09	[0.62; 1.92]	0.771	1.35	[0.67; 2.72]	0.401	1.37	[0.71; 2.66]	0.347
Low middle	1.42	[0.85; 2.37]	0.175	1.72	[0.79; 3.76]	0.176	1.66	[0.80; 3.44]	0.176
Low	1.54	[0.86; 2.73]	0.145	1.70	[0.68; 4.24]	0.252	1.71	[0.73; 3.99]	0.216
***Intermediate factors***									
**Mother’s age**									
15–24 years				1.00			1.00		
25–34 years				0.98	[0.43; 2.21]	0.953	1.06	[044; 2.58]	0.894
35–44 years				1.72	[0.49; 6.07]	0.399	1.84	[0.48; 7.06]	0.375
45–49 years				**0.13**	**[0.02; 0.80]**	**0.027**	0.16	[0.02; 1.02]	0.053
**Marital status**									
Married/in a union				1.00			1.00		
Never married				1.53	[0.38; 6.13]	0.547	1.57	[0.37; 6.65]	0.538
Divorced/separated/widowed				1.06	[0.53; 2.14]	0.866	1.14	[0.56; 2.33]	0.714
**Number of antenatal visits during most recent pregnancy**									
None				1.00			1.00		
1–3				1.12	[0.68; 1.84]	0.647	1.04	[0.62; 1.75]	0.880
4 +				0.75	[0.41; 1.37]	0.346	0.71	[0.36; 1.34]	0.290
Don’t know				0.47	[0.14; 1.56]	0.214	0.43	[0.13; 1.42]	0.166
**Mother’s age at child’s birth**									
20–34 years				1.00			1.00		
Less than 20 years				0.90	[0.45; 1.82]	0.777	0.98	[0.46; 2.05]	0.950
35–49 years				0.87	[0.34; 2.23]	0.770	0.92	[0.34; 2.51]	0.867
**Birth interval**									
First birth				1.00			1.00		
0–23 months				1.62	[0.79; 3.34]	0.191	5.25	[0.46; 60.58]	0.183
24–47 months				1.35	[0.68; 2.67]	0.388	4.49	[0.41; 49.11]	0.217
48 + months				0.60	[0.29; 1.23]	0.164	1.88	[0.17; 21.05]	0.606
**Number of living children in the household**									
1–2				1.00			1.00		
3–4				0.71	[0.36; 1.38]	0.310	0.83	[0.39; 1.77]	0.183
5 +				0.98	[0.46; 2.10]	0.962	1.24	[0.48; 3.23]	0.655
**Place of delivery**									
Home				1.00			1.00		
Health facility				1.14	[0.67; 1.93]	0.635	1.15	[0.69; 1.93]	0.588
**Had postnatal care within 42 days of delivery**									
No				1.00			1.00		
Yes				0.90	[0.60; 1.36]	0.617	0.85	[0.56; 1.29]	0.447
**Mother with no media exposure**									
No media exposure				1.00			1.00		
Some media exposure				0.87	[0.39; 1.91]	0.722	0.82	[0.39; 1.73]	0.604
**Mother listens to radio at least once a week**									
No				1.00			1.00		
Yes				0.98	[0.44; 2.17]	0.950	0.99	[0.47; 2.11]	0.983
**Mother watches TV at least once a week**									
No				1.00			1.00		
Yes				0.50	[0.13; 1.84]	0.293	0.51	[0.15; 1.75]	0.284
**Mother reads a journal at least once a week**									
No				1.00			1.00		
Yes				0.94	[0.44; 1.99]	0.863	0.93	[0.40; 2.15]	0.860
**Water source**									
Improved				1.00			1.00		
Not improved				0.65	[0.38; 1.12]	0.119	0.65	[0.39; 1.10]	0.106
**Toilet**									
Improved/not shared				1.00			1.00		
Not improved/shared				1.16	[0.44; 3.09]	0.762	1.11	[0.40; 3.11]	0.844
***Immediate factors***									
**Birth order**									
1							1.00		
2–3							0.33	[0.03; 3.65]	0.366
4–5							0.21	[0.02; 2.33]	0.205
6 +							0.25	[0.02; 2.91]	0.267
**Birth size**									
Average							1.00		
Very small							**8.16**	**[1.38; 48.34]**	**0.021**
Small							**3.44**	**[1.49; 7.94]**	**0.004**
Don’t know							1.34	[0.35; 5.06]	0.667
**Diarrhea in last 2 weeks**									
No							1.00		
Yes							1.30	[0.87; 1.94]	0.196
**Results of the Goodness of fit test**		F = 0.686	0.721		F = 1.216	0.285		F = 0.802	0.615

Model 1: Adjusted for child’s age, sex, and distal factors (province, residence, mother’s education, mother’s working status and wealth quintile)

Model 2: In addition to model 1, adjusted intermediate factors (mother’s age, marital status, number of antenatal visits, mother’s age at birth, birth interval, number of living children, place of delivery, postnatal care, mother’s media exposure, mother listens to radio, mother watches TV, mother reads journal, water source, toilet)

Model 3: In addition to model 2, adjusted for immediate factors (birth order, perceived birth size, diarrhea within 2 past weeks)

*OR* Odds ratio, *aOR* Adjusted odds ratio, *CI* Confidence interval

### Robustness checks of results

We checked the robustness of our results. We used the “svy” command prefix in all analyses to account for the complex survey design of the study used by the ASSP project. We assessed the fit of our final models using the “svylogitgof” which is used to estimate the goodness of fit when working on survey data [32]. We did not find evidence of lack of fit for all our final models since the *P* values yielded were all greater than 0.05.

## Discussion

This study aimed to determine the prevalence and risk factors associated with stunting, wasting, and underweight in children under-five years in four provinces of the DRC. Looking at the province level, we found that prevalence of stunting was 40.5%, 48.5%, and 32.7%, in Equateur, Kasai occidental, and Maniema/Oriental provinces respectively. Prevalence of wasting was 7.19%, 7.69%, and 9.68%, in Equateur, Kasai occidental, and Maniema/Oriental provinces respectively. Prevalence of underweight was 20.63%, 26.30%, and 14.16%, in Equateur, Kasai occidental, and Maniema/Oriental provinces respectively. These regional estimates may be helpful in informing the local, community-based interventions. Overall, we found that the prevalence of stunting, underweight, and wasting was 42.7%, 21.9%, and 8.2% respectively. These proportions are very similar to national estimates from the 2013–14 DRC’s DHS survey which found that 43% of children under-five were stunted, 23% were underweight and 8% were wasted. However, we cautiously make this comparison due to our sample not being nationally representative.In 2017–18, the stunting prevalence in DRC had fallen slightly to 41.8%, going from 44.4% in 2001, which shows a non-substantial decline of that indicator. However, the prevalence of wasting went from 15.6% in 2001 to 6.5% in 2017–18, which is a significant reduction [9]. Those declines could be explained by the country’s efforts to tackle undernutrition in preceding years. Indeed, DRC’s government adopted the National Nutrition Policy in 2013 implemented by the PRONANUT (National Nutrition Program). The policy aims at promoting best practices of exclusive breastfeeding of children aged less than 6 months, home fortification of complementary foods for children aged 6–23 months, interventions to improve the nutrition of pregnant and lactating women, the fight against micronutrient deficiency (Vitamin A, Iron, Iodine and Zinc), and early detection and management of childhood illness including acute malnutrition [33]. It also plans to reduce the prevalence of stunting by 50% and overall acute malnutrition below 10% by 2023 [9].

We also found that child’s age was a risk factor associated with stunting and underweight in children aged 0–23 months and overall children aged 0–59 months. However, it was not a risk factor of of wasting. There were no sex differences for the three indicators of undernutrition. The risk of stunting and underweight increased after the age of 6 months and became significant at 12 months and above. This finding may be because children aged 0–5 months are exclusively breastfed per the country’s policy and transition from exclusively being breastfed to complementary feeding after 6 months. Achieving good nutrition during this transition period may be challenging in a country experiencing food insecurity. Older age associated with higher rates of stunting has been also reported in studies conducted in Tanzania [12] and Burundi [14], two neighboring countries of DRC and in Pakistan [34]. However, higher risks of stunting were found among male children in the aforementioned studies, while we found no association between sex and stunting. In our study, girls had lower levels of wasting and underweight though the associations were not significant. Similar results were found for underweight in studies conducted in Tanzania [13] and Ghana [11].

At the distal level, we found that risks of stunting were higher among children aged 0–23 months and overall children aged 0–59 months for children who had mothers with low levels of education. This finding was also reported in studies conducted in Tanzania [12], Burundi [14], Ethiopia [35] and Bangladesh [20]. This highlights the importance of education of girls as a strategy to tackle stunting. Also, it has been demonstrated that parental education has a positive consequence on healthcare choices such as vaccination, family planning, and vitamin A supplementation [36]. Moreover, higher education is generally recognized to lead to higher income which may allow parents to afford healthcare services and sufficient food for their children [37]. In our study, we also found that children who had mothers who worked in the 12 months before the survey were at significant higher risk of stunting (in children aged 0–23 months and 0–59 months) and underweight (children 0–59 months). A possible explanation may be that those women did not have sufficient time to take care of their children because of their work schedule, though we cannot assert the type of work they were involved in. Also, they may have been a sole supporter of the child, which obliged them to work to provide for their child. Indeed, a study demonstrated that being a single mother was associated with stunting in DRC [38]. Regarding the provinces, we found higher odds of stunting and underweight in the province of Kasai occidental compared to the province of Equateur. Higher odds of stunting in the province of Kasai occidental were also found in the two studies conducted in DRC using the 2007 [17] and 2013–14 [15] DHS surveys. Artisanal diamond mining is the principal livelihood activity in that province, and few people rely on food production through agriculture. Most food in the province is imported, possibly decreasing access to food [15, 17]. A recent nation-wide study using the DRC’s MICS6 (2017–18) has demonstrated that women’s maternal health-seeking behaviors (MHSB: antenatal care, institutional delivery, and postnatal care) was worse in the regions of Kasai (i.e., Kasai occidental split in Kasai and Kasai Central, Kasai Oriental, Sankuru and Lomami). In 2016, violence erupted in the region, attacking health facilities and thus hindering women from having MHSB [39]. This situation can worsen undernutrition that strikes the region as it was shown that the use of MHSB significantly decreases the risk of malnutrition [40]. A special attention should be paid to the Kasai regions and projects targeting agriculture and MSHB should be envisioned.

At the intermediate level, we found that children aged 0–59 months who were born in a health facility were more prone to be stunted than their counterparts who were born at home. This finding contradicts what Nkurunziza et al. found in Burundi [14], Abuya et al. in Kenya [41] and Amaha et al. in Ethiopia [35], where children who were born at home were more at risk of stunting. Theoretically, women who deliver in a health facility are hypothesized to often use health care services, and therefore are more informed on good health care for their children. In our study, this unexpected result could be because those who deliver in health facilities may be people that have health issues. We also found that children aged 0–59 months who were born to mothers aged 35–49 years were more likely to be wasted compared to children who were born to mothers aged 20–34 years. Similar results were found in a study conducted in five Asian countries using DHS surveys [42]. In DRC, prevalence of birth control is low in older women [8], implying that those older women may have many children to care for, and thus at risk of undernutrition. However, these results contradict those found in a study conducted in Ghana [43] where it was shown that children born to younger mothers were more at risk of wasting. Younger mothers may be less experienced than older mothers in childcare and are more at risk of intrauterine growth restriction, preterm birth and low birth weight due to competition of nutrients between the growing mother and the fetus [44]. More generally, we found few associations between intermediate factors and undernutrition in models 3. For example, none of the undernutrition indicators was associated with the type of drinking water source or sanitation facilities. This may be because responses to the survey were self-reported. Similar results were found for stunting in cross-sectional studies conducted in DRC [15] and Burundi [14] and for the three undernutrition indicators in Ghana [11, 45]. Nevertheless, a cross-sectional study conducted in Papua New Guinea demonstrated that unimproved toilets were associated with underweight and stunting and that unimproved water source was associated with underweight [46]. Other cross-sectional studies conducted in Uganda [47] and Tanzania [12] showed that children using non-improved water sources were significantly more at risk of stunting than their counterparts who used improved water sources. Besides, we did not find significant associations between media factors (mother has no access to media, reads journal at least once a week, listens to radio at least once a week, watches TV at least once a week) and undernutrition. This may be due to low access to media factors in our sample. Same results were found for stunting in Tanzania [12]. Also, a cross-sectional study conducted in Pakistan did not find significant association between women having access to media and the three indicators of undernutrition [34]. However, Akombi et al. found that in Nigeria, children who had mothers who did not read a journal or did not watch TV were at higher risks of stunting [19].

At the immediate level, children perceived by their mothers to be very small or small at birth were significantly more prone to be stunted and underweight compared to those who were perceived to be average at birth. This supports results found in Tanzania [12], Burundi [14] and Nigeria [19]. Given the high prevalence of low birth weight (9.5%) in DRC [48], policies should focus on prevention of intrauterine growth restriction and preterm birth which predispose children to having low birth weight. This could be a pathway to tackle undernutrition in children from DRC. Finally, in our study, breastfeeding status of the youngest among children aged 0–23 months was significantly associated with wasting in the breastfeeding + water category, compared to the exclusively breastfeeding category. Although exclusively breastfeeding is widely accepted in DRC, 65% of children still receive something other than breastmilk by the age of 2 to 3 months [49]. The negative consequences of complementary breastfeeding before 6 months has been reported in a study in Malawi [50]. Educational programs related to the dangers of supplemental water during the first six months of life specifically should be implemented.

### Strengths and limitations

We included a large set of potential explanatory variables and employed robust statistical models using the sampling weights to account for the effects of clustering and stratification of data. Another strength of this study is the use of the UNICEF framework to identify risk factors associated with undernutrition in children. Province was one of distal factors, therefore we did not stratify our analysis by province. Nevertheless, this study suffered from the cross-sectional design of the survey that does not allow us to establish the temporality needed for causal inference. Also, women were interviewed concerning children they gave birth to in the 5 years preceding the survey, which may be a source of recall bias. While it would have been useful to break down the province information into the current administrative boundaries, this was not possible as the sampling strategy was based on the older administrative boundary definition and the stratified clustering design used the aggregated populations as the base population. Finally, our study covered only 4 provinces out of 11 that constituted the DRC (at the time when data were collected), which may not allow us to generalize our results to the whole country.

### Nutritional policy considerations

Underlying drivers of malnutrition differ from one country to another. Thus, designing a local setting-specific and context-sensitive nutrition program in an evidence-based manner is the key to ensuring more effective and efficient interventions. Two categories of nutrition interventions have been used to reduce undernutrition: nutrition-sensitive interventions and nutrition-specific. Nutrition-sensitive interventions are those that act on underlying determinants of nutrition, e.g., water, sanitation and hygiene, schooling, child protection, early child development, maternal mental health, agriculture and food security, health and family planning services, social safety nets, and women’s empowerment [51]. Nutrition-specific interventions target immediate determinants of nutrition of the fetus and the child, namely supplementation in vitamin A and zinc, exclusive breastfeeding, dietary diversity promotion, and food fortification [52]. The results of this study indicate the need for intervention at distal, intermediate, and immediate levels. At the distal level, emphasis should be placed on education of the youth, especially young girls, women empowerment, and sensitization on agricultural practices and MHSB with a special attention in the Kasai provinces. At the intermediate level, a focus should be put on family planning and maternal health in general. Finally, at the immediate level, there is a need of promoting exclusive breastfeeding during the first six months through education of mothers and preventing intrauterine growth restriction and preterm birth.

## Conclusion

Rates of undernutrition in DRC are high. We have identified some risk factors associated with stunting, wasting and underweight. These factors may vary per each undernutrition indicator. We found that province, education level, mother’s working status, place of delivery, age at birth, perceived birth size and breastfeeding status were modifiable risk factors associated with undernutrition. Our study highlights the importance to intervene at the distal, intermediate, and immediate levels.

## Data Availability

Data used in this analysis are available from IMA World Health DRC, but restrictions apply to the availability of these data, which were used under license for the current study, and so are not publicly available. Data are however available from the authors upon reasonable request and with permission of IMA World Health DRC.

## Abbreviations

aOR: Adjusted Odds Ratio
ASSP: Accès aux Soins de Santé Primaires
BCZ: Bureau Centrale de la Zone de Santé
CI: Confidence Interval
DFID: Department for International Development
DHS: Demographic and Health Surveys
DRC: Democratic Republic of Congo
ECZS: Equipe Cadre de la Zone de Santé
HA: Health Area
HAZ: Height-for-Age Z-scores
HZ: Health Zone
IMA: Interchurch Medical Assistance
IRB: Institutional Review Board
MICS: Multiple Indicators Cluster Surveys
OR: Odds Ratio
PRONANUT: Programme National de Nutrition
SD: Standard Deviation
SSA: Sub-Saharan Africa
TV: Television
UK: United Kingdom
UNICEF: United Nations Children’s Fund
VIF: Variance Inflation Factor
WAZ: Weight-for-Age Z-scores
WHO: World Health Organization
WHZ: Weight-for-Height Z scores
Z-BMI: Body Mass Index Z-scores
